# The Sphingosine and Acyl Chains of Ceramide [NS] Show Very Different Structure and Dynamics That Challenge Our Understanding of the Skin Barrier

**DOI:** 10.1002/anie.202003375

**Published:** 2020-08-07

**Authors:** Oskar Engberg, Andrej Kováčik, Petra Pullmannová, Martin Juhaščik, Lukáš Opálka, Daniel Huster, Kateřina Vávrová

**Affiliations:** ^1^ Institute of Medical Physics and Biophysics University of Leipzig Härtelstr. 16–18 04275 Leipzig Germany; ^2^ Skin Barrier Research Group Faculty of Pharmacy in Hradec Králové Charles University Akademika Heyrovského 1203 50005 Hradec Králové Czech Republic; ^3^ Department of Chemical Science Tata Institute of Fundamental Research Dr. Homi Bhabha Road, Colaba Mumbai 400 005 India

**Keywords:** chain conformation, lipids, membranes, nanostructure, NMR spectroscopy

## Abstract

The lipid phase of the uppermost human skin layer is thought to comprise highly rigid lipids in an orthorhombic phase state to protect the body against the environment. By synthesizing sphingosine‐d_28_ deuterated N‐lignoceroyl‐d‐erythro‐sphingosine (ceramide [NS]), we compare the structure and dynamics of both chains of that lipid in biologically relevant mixtures using X‐ray diffraction, ^2^H NMR analysis, and infrared spectroscopy. Our results reveal a substantial fraction of sphingosine chains in a fluid and dynamic phase state at physiological temperature. These findings prompt revision of our current understanding of the skin lipid barrier, where an extended ceramide [NS] conformation is preferred and a possible domain structure is proposed. Mobile lipid chains may be crucial for skin elasticity and the translocation of physiologically important molecules.

## Graphical abstract

**Dynamic chains in a rigid matrix**: While the sphingosine chains of ceramide molecules in the outermost human skin layer were originally assumed to be fully rigid, spectroscopic data reveals that they display a complex dynamic behavior. These new insights will help fine‐tune the molecular model of the skin lipid barrier.

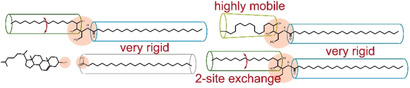

For several decades, the lipid phase of the uppermost human skin layer, the *stratum corneum* (SC), has been described as a continuous assembly of highly ordered and rigid lipids.^1^ The SC is crucial for survival on dry land as it protects the body against excessive loss of water and penetration of harmful xenobiotics. The SC lipids consist of an approximate equal molar mixture of ceramides (Cer) of varying polar head structures and chain lengths, free fatty acids, and cholesterol (Chol). Unlike phospholipid bilayers, the SC lipids form multilamellar assemblies with very low water content of 1–2 water molecules per lipid headgroup.^2^ Most structural insight comes from X‐ray and neutron diffraction,^3^ ^2^H and ^13^C MAS NMR,^4^, ^5^, ^6^ and Fourier transform infrared (FTIR) spectroscopy.^7^, ^8^ At physiological skin temperature (32 °C), these methods have reported a coherent model describing the SC lipid phase as densely packed orthorhombic/hexagonal layers forming either a short (SPP) or a long periodicity phase (LPP).^3^ Dynamic lipids are only detected at high/unphysiological temperatures.^5^, ^9^, ^10^

Considering the highly dynamic nature of physiological lipid membranes,^11^ it is remarkable that the skin lipids form a highly rigid phase. Consequently, this phase state has been pronounced to represent the basis for the skin's barrier function. However, the biophysical methods used to study the SC may impose some dynamical bias. Diffraction techniques are sensitive to ordered structures and thus most suited to describe the rigid phase of lipid layers.^3^, ^10^ Solid‐state ^2^H NMR spectroscopy relies on isotopic labeling, limited to specific molecular segments as provided by chemical synthesis. Thus, the information is constrained to the labeled moieties and only provides a restricted and possibly biased view on the dynamic behavior of the molecules. In SC research, ^2^H labeling is available for the Cer acyl chains, the fatty acids, and Chol, which were shown as highly rigid in many studies.^4^, ^5^, ^12^ Methods that do not require isotopic enrichment (^13^C MAS NMR^6^ and FTIR^7^, ^8^ spectroscopy) are confronted with dynamic range problems as the highly intense NMR peaks or vibrational bands of the chain methylene groups render detection of low‐intensity signals very difficult. These segments, however, could feature an alternative dynamic signature. In that regard, the recent observations that the oleic^9^ or linoleic acyl chain^10^, ^13^ moieties of the ultra‐long‐chain Cer[EOS] in SC mixtures are isotropically mobile in SC mixtures at physiological temperature while all other lipids remained orthorhombic and rigid caused attention.

These new findings prompt the question whether also other segments of highly abundant Cer molecules in the SC that have eluded detailed investigation so far could possibly exhibit higher mobility. The most prominent structural moiety that has not been studied up to now is the sphingosine backbone. To close this crucial gap, we synthesized the most canonical Cer molecule Cer[NS] with a perdeuterated sphingosine and studied the dynamic properties of that chain. Up to now, only the acyl chain moiety of Cer[NS] has been deuterated and all results about its mobility and phase state have been derived from such species.^5^, ^9^

Cer[NS]‐sph‐*d*_28_ was constructed by cross‐metathesis of perdeuterated pentadec‐1‐ene (**3**) with protected C_5_‐sphingosine (**5**) (Scheme 1). First, perdeuterated pentadecan‐1‐ol (**1**) was dehydrated by the Grieco protocol;^14^ the yields of selenide (**2**) formation (96 %) and its H_2_O_2_ oxidation to pentadec‐1‐ene‐*d*_30_ (**3**) (96 %) were comparable to those reported for unlabeled compounds.^14^ This facile generation of deuterated terminal alkenes opens further possibilities for the synthesis of specifically deuterated compounds for applications in the life sciences,^15^ as terminal alkenes are ideal substrates for numerous chemical transformations.^16^ Cross‐metathesis of pentadec‐1‐ene‐*d*_30_ (**3**) with protected C_5_‐sphingosine (**5**), prepared by vinylation of (*S*)‐Garner aldehyde (**4**), proceeded in 37±5 % yield (*n=*3) to provide the protected sphingosine‐*d*_28_ (**6**). This yield is significantly lower than that of the metathesis of unlabeled pentadec‐1‐ene (57±1 %; *n=*3, *p*<0.05), which may be consistent with a secondary kinetic isotope effect resulting from the sp^3^ to sp^2^ hybridization change of the metallacycle carbons during breakup.^17^ Sphingosine‐*d*_28_ (**6**) was deprotected and acylated to yield the final Cer[NS]‐sph‐*d*_28._ Cer[NS]‐acyl‐*d*_47_ with a deuterated C_24_‐acyl chain was prepared by acylating sphingosine with lignoceric acid‐*d*_47_.^18^

**Scheme 1:**
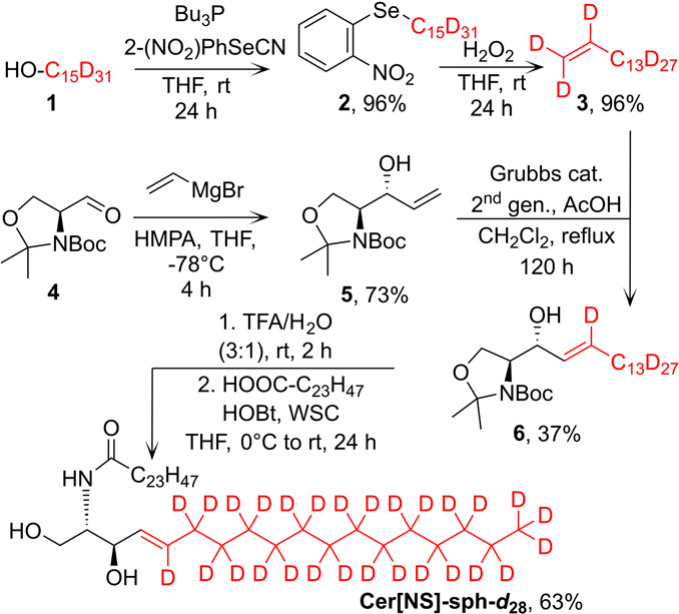
Chemical synthesis of deuterated Cer[NS]‐sph‐*d*_28_.

With the new compound in hand, we prepared two standard mixtures of Cer[NS], lignoceric acid (LA), and Chol at molar ratios of 1:1:1 and 1:1:0.45. The latter was prepared to avoid the crystalline Chol phase that had been observed previously.^19^, ^20^ This model represents the major lipid classes in correct proportions and, although it does not fully capture the structural heterogeneity of ceramides and fatty acids as this is technically not possible (these lipids comprise over 1000 chemically distinct molecules), it retains the characteristic very long saturated chains of these lipids. Our lipid models also mimic the multilamellar assemblies with dominant orthorhombic chain packing as shown by the X‐ray diffraction experiments that were carried out (Supporting Information, Figure S1) on non‐oriented samples after NMR measurements. Well‐resolved Bragg peaks up to the 6^th^ order were obtained, providing repeat distances of 53.8 and 53.9 Å, respectively, characteristic for the SPP.^3^ The high Chol sample also showed a separated Chol phase with a *d*_(001)_ reflection corresponding to 34.5 Å.^3^ In the wide‐angle region, both samples showed two reflections corresponding to distances between lattices of *d=*4.1 and 3.7 Å, characteristic for orthorhombic chain packing.^3^

Next, the Cer[NS] in the mixtures was studied by solid‐state ^2^H NMR spectroscopy (Figure 1). Cer[NS] was deuterated either in the acyl chain (Cer[NS]‐acyl‐*d*_47_) or in the sphingosine moiety (Cer[NS]‐sph‐*d*_28_). Thus, the structure and dynamics of each hydrocarbon chain could be studied individually, providing a complete overview of the thermotropic phase behavior of Cer[NS] in Cer[NS]/LA/Chol mixtures. ^2^H NMR spectra of high quality were detected, indicating very homogeneous preparations.

**Figure 1:**
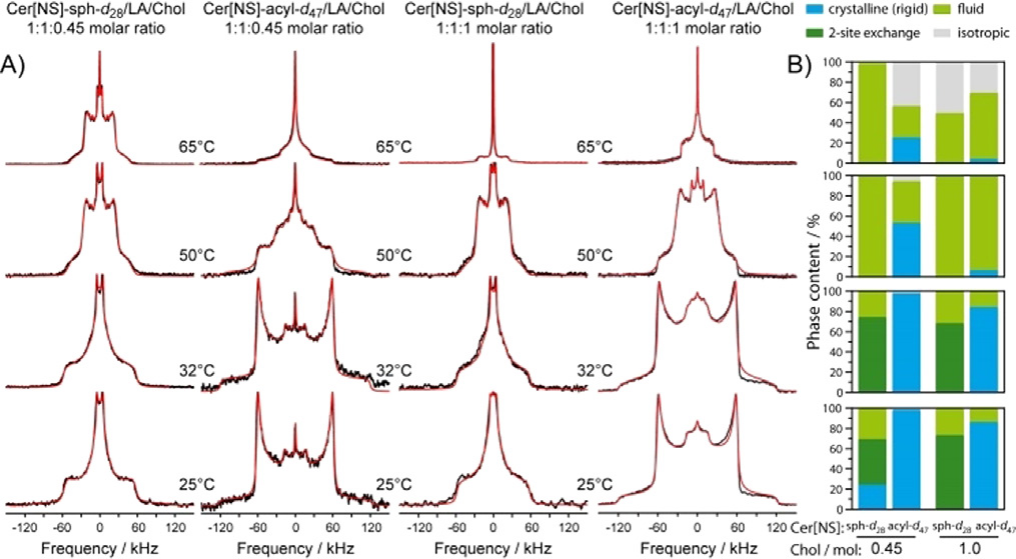
A) Static ^2^H NMR spectra (black) of the SC lipid model mixture composed of Cer[NS]/LA/Chol at molar ratios of 1:1:0.45 (left two columns) and 1:1:1 (right two columns) at various temperatures. The first and third column display the ^2^H NMR spectra of the Cer[NS]‐sph‐*d*_28_ (deuterated sphingosine‐*d*_28_). Columns 2 and 4 show the NMR spectra of the Cer[NS]‐acyl‐*d*_47_ (perdeuterated lignoceroyl‐*d*_47_). Simulated spectra are shown in red. The multilamellar lipid preparations contained 50 wt % buffer (0.1 m MES, 0.1 m NaCl, 5 mm EDTA, pH 5.4). B) Relative proportions of the individual phases observed in the mixtures.

From the ^2^H NMR spectra, the structure and dynamics of the two Cer[NS] chains were derived. We used numerical lineshape simulations to determine the structural and dynamical parameters of the chains and quantify the relative proportions of the phases formed.^4^, ^5^ These simulations reproduce spectral lineshapes for rigid (all‐*trans*) lipid chains assuming a static electric field gradient (EFG) tensor, or consider the effect of motions partially averaging the EFG tensor relevant for lipid chains undergoing two‐site jumps, *trans*–*gauche* isomerization, or isotropic motions (vide infra). The Cer[NS]‐acyl‐*d*_47_ lignoceroyl chain showed a predominant orthorhombic phase state at 25 and 32 °C in both mixtures. Such NMR spectra are obtained for rigid all‐*trans* acyl chains.^5^, ^12^ Only the methyl groups undergo three‐site hopping, reducing the quadrupolar splitting by 1/3.^21^ In stark contrast, the Cer[NS]‐sph‐*d*_28_ sphingosine moiety shows much different NMR spectra featuring two contributions. The first represents an intensity distribution characteristic for the gel phase known from phospholipid membranes below the main phase transition.^22^ Such ^2^H NMR spectra have been described for the C_16_ acyl chains of Cer[NS] assuming a two‐site exchange model.^5^ In the two‐site exchange model, the all‐*trans* chains interchange about their long molecular axis with a jump angle of 180°. Indeed, this model explains the broad base of the ^2^H NMR spectra of sphingosine‐*d*_28_. In addition, the NMR spectra contain a narrow component that was simulated by a superposition of Pake doublets scaled by relatively low order parameters. This combination of powder line shapes exactly explains the experimental NMR spectra (Figure 1 A).

From the simulations of the NMR spectra, the relative proportions of each phase were determined (Figure 1 B and Table S1). At 25 °C and 32 °C, the high and the low Chol mixtures behave very similarly. At both temperatures, the majority (84–98 %) of the lignoceroyl‐*d*_47_ acyl chain of Cer[NS] forms an orthorhombic phase in agreement with previous reports.^4^, ^5^, ^9^ At high Chol, a very small fluid fraction is observed. However, completely different phases were observed for the deuterated sphingosine‐*d*_28_. The NMR spectra indicate major contributions (between 45 % and 74 %) from sphingosine chains that are subject to two‐site exchange processes. Very surprisingly, a substantial amount (between 26 % and 32 %) of the sphingosine is highly fluid, as indicated by very narrow ^2^H NMR spectral components. In this state, the sphingosine chains are very disordered, undergoing motions with large amplitudes on the order of 40°. While the orthorhombic lignoceroyl moieties and the sphingosine chains undergoing two‐site exchange exhibit rigid C−^2^H bond vectors that do not exhibit any fluctuations at all, the fluid sphingosine chains show an order parameter profile indicative of large‐amplitude motions for the methylene segments (Figure 2 A). Order parameters decrease very steeply from the upper chain segments to the chain end, as observed for inverse hexagonal phases of lipid membranes,^23^ concomitant with increased spatial requirements of the fluid sphingosine chains.

**Figure 2:**
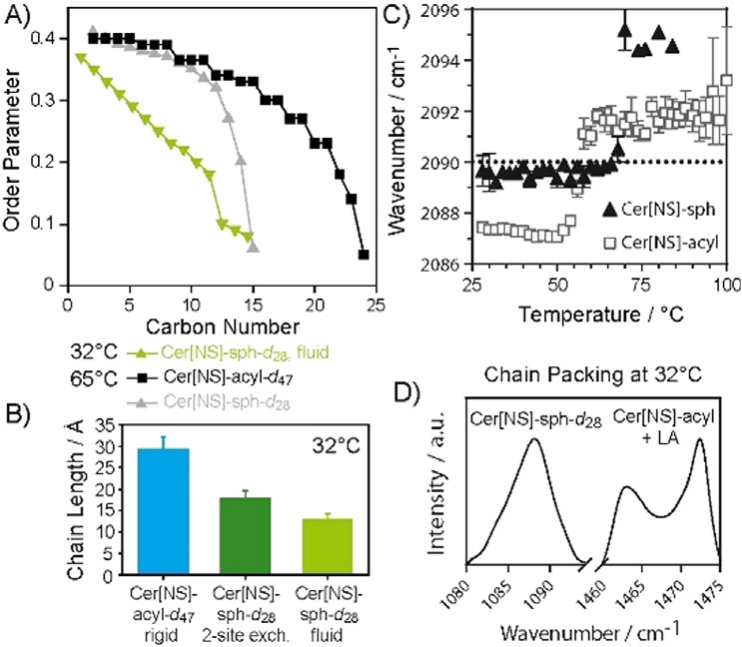
Order parameter profiles (A), projected chain lengths (B) of the Cer[NS] from NMR measurements. IR wavenumbers for the methylene symmetric stretching bands (C) and IR spectra of the CD_2_ and CH_2_ scissoring bands (D) of SC model mixtures (Cer[NS]/LA/Chol, 1:1:1 molar ratios; 0.13 mol Chol‐sulfate for FT measurements).

It is very instructive to compare the geometrical parameters of the lignoceroyl and the sphingosine chains of Cer[NS] at physiological skin temperature (32 °C). The lignoceroyl chain is in an all‐*trans* state (length *l*=29.2 Å). The sphingosine chains undergoing two‐site exchange are also all‐*trans* (*l*=17.8 Å). In contrast, the fluid sphingosine chains are disordered by fast *trans*–*gauche* isomerizations. Using analytical models,^24^ the average length of the dynamic sphingosine moiety was calculated from the order parameters. Rapid *trans–gauche* isomerizations lead to a decrease in the chain length to 13.0 Å for the high and 12.7 Å for the low Chol mixtures. Thus, each sphingosine chain contains on average 4.3 or 4.6 *gauche* defects at 32 °C (one *gauche* defect reduces the chain length by 1.1 Å).^25^ The lengths of chains of Cer[NS] subject to different molecular packing and dynamics are illustrated in Figure 2 B.

At 50 °C, both SC mixtures undergo restructuring. All NMR spectra of the sphingosine as well as the lignoceroyl moieties indicate fluid phases. While the sphingosine of the Cer[NS] is completely fluid, the lignoceroyl chain is still found to be 53 % in the orthorhombic phase at low and 6 % at high Chol content. At 65 °C, the orthorhombic proportions of the lignoceroyl chains of Cer[NS] have almost completely disappeared. The dominant phase is the fluid state, characterized by ^2^H NMR spectra reminiscent of the liquid‐ordered phase observed for sphingomyelin in raft mixtures.^26^, ^27^ The sphingosine moiety is entirely fluid and/or isotropic. In the isotropic phase, the multilayer structure of the SC model is fully abolished but lamellar structures are formed again when the temperature is decreased. The ^2^H NMR spectra can be well simulated assuming axially symmetric reorientations of the molecules along their long axis and a gradient of molecular order that is decreasing towards the end of the chain. These assumptions lead to a perfect description of the ^2^H NMR spectra with order parameter profiles shown in Figure 2 A. The two chains feature similar order parameters for the upper half of the chain while order decreases steeper for the shorter sphingosine moiety towards the end of the chain. The presence of Chol leads to relatively large quadrupolar splittings observed under these conditions. At high Chol concentration, the projected average chain length for the lignoceroyl moiety on Cer[NS] is 23.3 Å, suggesting that on average 5.3 C−C bonds are in the *gauche* state. Similarly, the sphingosine moiety shows an average length of 14.7 with 2.8 *gauche* defects per chain. At low Chol content, for the lignoceroyl chain, the average chain length is 23.6 Å corresponding to 5.1 *gauche* defects. For the sphingosine moiety, we calculate an average chain length of 14.9 Å, which requires on average 2.7 *gauche* defects. Clearly, there is no chain length match between the lignoceroyl chain and the sphingosine moiety of Cer[NS]. At 80 °C, both deuterated chains of Cer[NS] are fully isotropic (Figure S2).

The lipid mixture consisting of Cer[NS]/LA/Chol (1:1:1 molar ratio, with 0.13 mol Chol‐sulfate, a minor SC component) was studied by FTIR spectroscopy. Figure 2 C shows that sphingosine is less ordered than the acyl chain at 32 °C, as indicated by almost by 2 cm^−1^ higher wavenumber of the CD_2_ symmetric stretching bands, consistent with the number of *gauche* defects seen by NMR. This wavenumber shift is rather large for SC lipids. Wavenumbers were increased by 0.4 cm^−1^ in patients with atopic dermatitis as compared to healthy individuals.^28^ The chain melting phase transitions of the sphingosine (56 °C) and lignoceroyl chains (68 °C) of Cer[NS] are clearly separated. Figure 2 D shows a well‐resolved CH_2_ scissoring doublet in the mixture with Cer[NS]‐sph‐*d*_28_ at 32 °C. This doublet arising from CH_2_ vibrational coupling indicates that the unlabeled chains (Cer acyl and LA) are orthorhombic and do not mix with the labeled sphingosine chains, as the coupling only occurs between the same isotopes. The orthorhombic doublet disappears at 37 °C,^18^ which agrees well with the orthorhombic–hexagonal transition found in human SC.^29^, ^30^ This pretransition is also consistent with the loss of the crystalline phase between 32 °C and 50 °C seen by NMR spectrscopy.

There are several important structural consequences for our current understanding of the SPP of the SC lipid barrier. 1) The two chains of Cer[NS] show a very different dynamic behavior in mixtures comprising major SC lipid classes. At physiological temperature, where the lipid phase is predominantly orthorhombic, about 1/3 to 1/4 of the sphingosine chains are highly dynamic featuring low order parameters caused by rapid *trans–gauche* isomerizations. Compared to all‐*trans* chains, such dynamic sphingosine moieties occupy a 36 % larger cross‐sectional area in the layers. Such an area increase in the tightly packed SC will create defects possibly responsible for the (limited) permeability of the outer skin layer. 2) Considering the very different dynamic states of the lipid chains of Cer[NS], a hairpin model of this Cer is unlikely. Both the fluid and the sphingosine chains in two‐site exchange require a symmetry axis for these motions that is parallel to the long axis of the molecule. Since no indications for any such dynamics of the lignoceroyl moiety were found in the ^2^H NMR spectra, the hairpin model of Cer[NS] in the SC layers has to be rejected. Even with an all‐*trans* lignoceroyl chain conformation, the required axially symmetric rotation of Cer[NS] would decrease the lignoceroyl order parameters to 0.5, which was clearly not observed. Only an extended conformation of Cer[NS], where the two chains can move independently, would agree with our experimental data. Even at elevated temperatures, we see very different dynamical features of the lignoceroyl and the sphingosine chains that would not agree with the hairpin conformation. 3) To avoid large packing defects caused by chain‐length mismatches of the dynamic and rigid chains at the interface between orthorhombic lignoceroyl and highly mobile sphingosine chains, it becomes likely that the lipid phase of the SC shows a domain structure. Since the highly mobile and fully extended sphingosine chains feature very different lengths, a random distribution of lipids would produce frequent hydrophobic mismatches that are energetically highly unfavorable. These situations are avoided by clustering mobile and rigid chains, respectively, minimizing the interface between the domains characterized by very different chain lengths. Such fluctuations in lipid distribution would also agree with modern models of fluid biological membranes.^26^, ^27^ Taken together, 4) with the new structural data reported here, we suggest a model of the SPP of the SC mixture as sketched in Figure 3. At skin temperature, the Cer molecules are in an extended conformation and highly mobile and highly rigid chain moieties are likely to cluster together, introducing heterogeneities and fluctuations to the lipid phase of the SC. Such mobile SC lipid chains may be important to maintain skin elasticity and enable translocation of physiologically important molecules, such as water, cytokines, and antimicrobial peptides, which is consistent with the current view on the SC sensory and defensive functions. Thus, with the current and two recent^9^, ^10^ reports on the high mobility of specific segments of Cer molecules in the SC, it appears that the models of the lipid structures of the outermost layer of human skin will have to be revised to consider more molecular disorder and dynamics of the lipids under physiological conditions.

**Figure 3:**
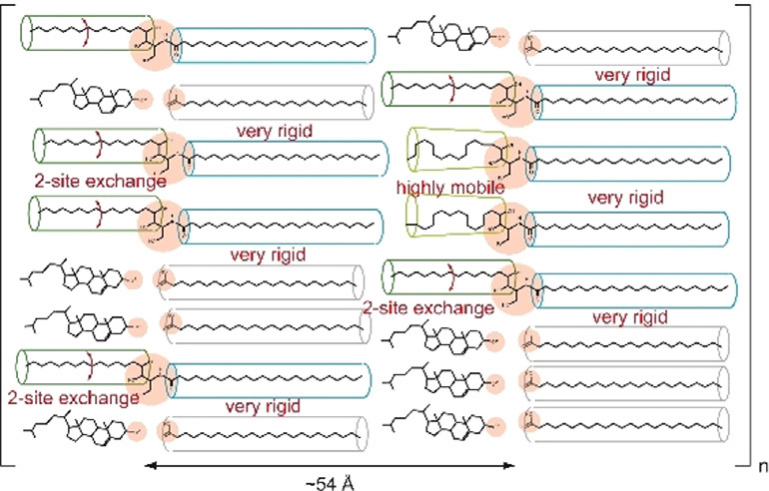
Model of the SPP of the SC mixture composed of an equimolar ratio of Cer[NS]/LA/Chol at physiological skin temperature.

## Conflict of interest

The authors declare no conflict of interest.

## Supporting information

As a service to our authors and readers, this journal provides supporting information supplied by the authors. Such materials are peer reviewed and may be re‐organized for online delivery, but are not copy‐edited or typeset. Technical support issues arising from supporting information (other than missing files) should be addressed to the authors.

Supplementary
