# Supplementary material for: The Sphingosine and Acyl Chains of Ceramide [NS] Show Very Different Structure and Dynamics That Challenge Our Understanding of the Skin Barrier
Source: Angew Chem Int Ed Engl. 2020 Aug 7;59(40):17383–7. doi: 10.1002/anie.202003375 (PMC7540555; doi:10.1002/anie.202003375)
Supplement: Supplementary file 1 — Supplementary [file ANIE-59-17383-s001.pdf]

Angewandte Chemie
Eine Zeitschrift der Gesellschaft Deutscher Chemiker

Supporting Information

The Sphingosine and Acyl Chains of Ceramide [NS] Show Very Different Structure and Dynamics That Challenge Our Understanding of the Skin Barrier

Oskar Engberg+, Andrej Kováčik+, Petra Pullmannová+, Martin Juhaščik, Lukáš Opálka, Daniel Huster, and Kateřina Vávrová**

anie_202003375_sm_miscellaneous_information.pdf

Table of Contents

1 Experimental Procedures 2
1.1 Materials 2
1.2 General experimental procedures and instrumentation 2
1.3 Synthetic procedures and NMR spectra 2
1.4 Sample preparation 14
1.5 Solid-state ^{2}H NMR spectroscopy 14
1.6 X-Ray diffraction 14
1.7 Infrared spectroscopy 15
2 Results 16
2.1 Supplementary Figure S1 16
2.2 Supplementary Figure S2 17
2.3 Supporting Table S1 17
3 References 19

1 Experimental Procedures

1.1 Materials

Cholesterol and lignoceryl-D-*erythro*-sphingosine (Cer[NS]) were purchased from Avanti Polar Lipids (Alabaster, AL, USA), 1-pentadecanol-d_{31} was purchased from C/D/N Isotopes (Pointe-Claire, Canada). Lignoceric acid (LA) and all solvents and other chemicals for the synthesis were purchased from Merck (Darmstadt, Germany) and used without further purification. Cer[NS]-acyl-d_{47} with uniform acyl chain perdeuteration was synthesized as described before.[1]

1.2 General experimental procedures and instrumentation

All reactions were performed under an argon atmosphere. Air and/or moisture-sensitive liquids were transferred via syringe. Organic solutions were concentrated by rotary evaporation (Büchi Rotavapor R-114, Flawil, Switzerland). Visualization of TLC plates was achieved by immersion in water solution of cerium (IV) sulfate, phosphomolybdic and sulfuric acid, followed by heating at 230°C. The structure and purity of the prepared structures were confirmed by ^{1}H and ^{13}C NMR spectrometry (VNMR S500 NMR spectrometer; Palo Alto, CA, USA). Chemical shifts (δ) are reported in parts per million (ppm) and were indirectly referenced to tetramethylsilane via the solvent signal. Data are presented as follows: chemical shift, multiplicity (s = singlet, d = doublet, t = triplet, m = multiplet), coupling constant (J, Hz) and integration. IR spectra were recorded on a Nicolet 6700 in the ATR mode (Thermo Scientific, Waltham, MA, USA). Melting points were determined using a Kofler hot-stage microscope and are uncorrected. Mass spectrometry was measured on an LCQ Advantage Max (Thermo Finnigan, San Jose, USA) equipped with an APCI source. Elemental analysis was performed on a Vario Micro Cube Elemental Analyzer (Elementar Analysensysteme GmbH, Hanau, Germany).

1.3 Synthetic procedures and NMR spectra

(2-Nitrophenyl)(pentadecyl)selane-d_{31} (2)

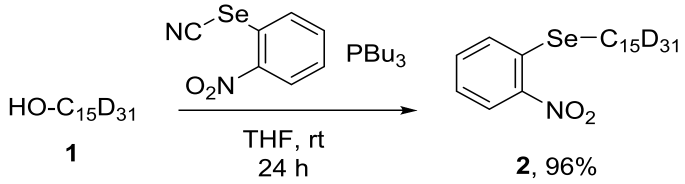

To a solution of 1-pentadecanol-d_{31} **1** (398 mg, 1.54 mmol) and 2-nitrophenyl selenocyanate (524 mg, 2.3 mmol) in dry THF (20 mL), tributylphosphine (0.6 mL, 2.3 mmol) was added and the reaction mixture was stirred under argon atmosphere at room temperature overnight. The reaction mixture was then concentrated under reduced pressure and purified by column chromatography using hexane/Et_2O (10:1) as a mobile phase to obtain yellow crystals **2** (842 mg, 96%). TLC: hexane/Et_2O (3:1), R_f = 0.71. M. p. 53–56°C. ^{1}H NMR (500 MHz, $CDCl_3$): δ = 8.32 – 8.27 (m, 1H), 7.57 – 7.46 (m, 2H), 7.35 – 7.29 (m, 1H) ppm. ^{13}C NMR (125 MHz, $CDCl_3$): δ = 146.94, 133.98, 133.43, 128.38, 126.45, 125.17, 28.44 ppm. IR: ν_{max} 2192, 2089, 1509, 1333, 1308, 1099, 721 cm^{-1}.

^{13}C NMR (125 MHz, $CDCl_3$)

(2-Nitrophenyl)(pentadecyl)selane-d_{31} **(2)**

^{1}H NMR (500 MHz, $CDCl_3$)

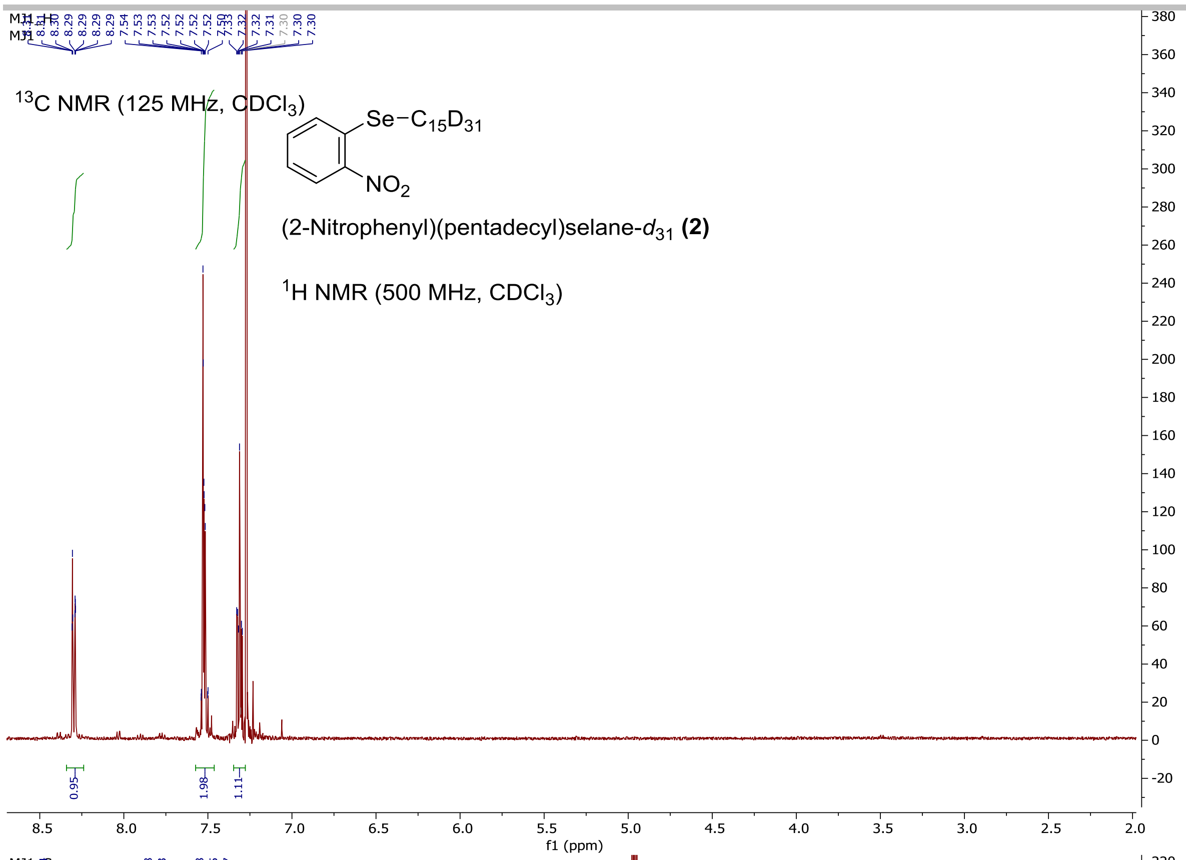

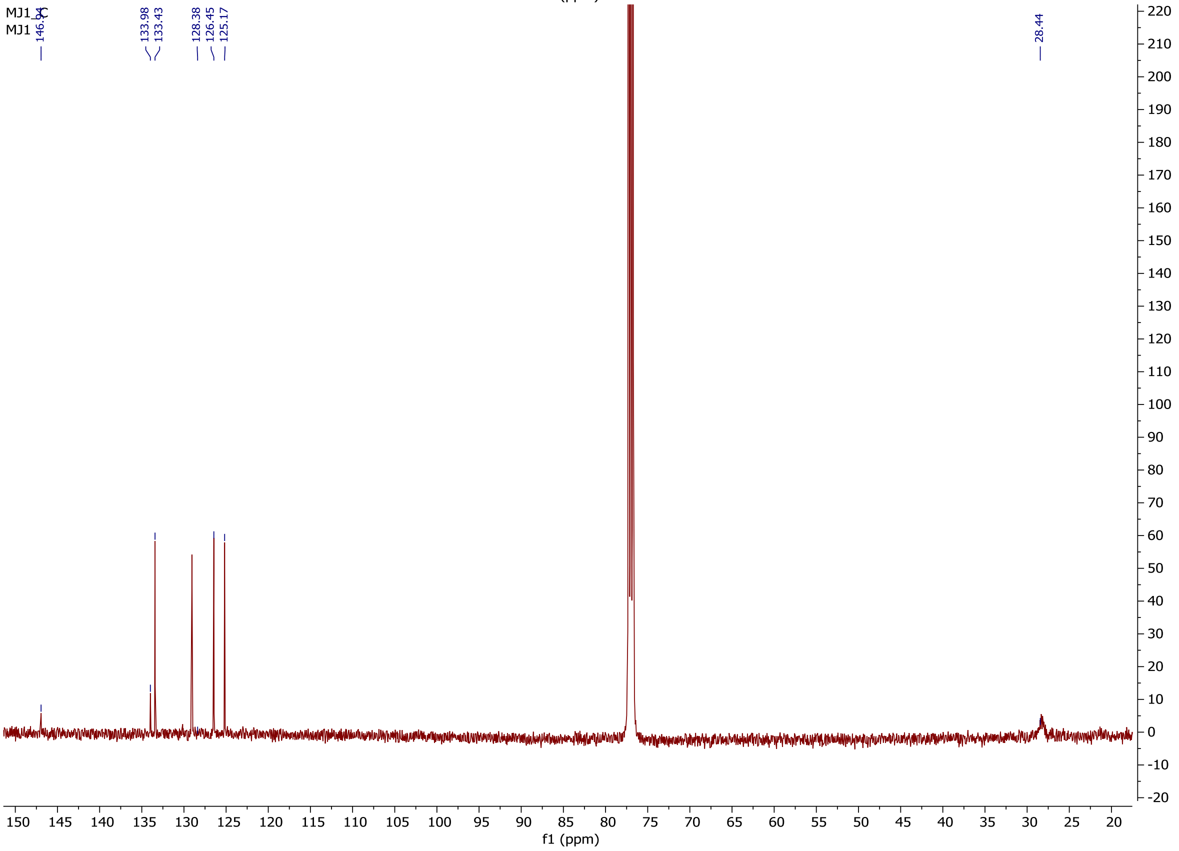

Pentadec-1-ene-d_{30} (3)

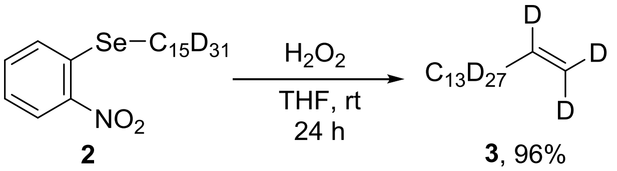

To a solution of **2** (511.5 mg, 1.153 mmol) in dry THF (15 mL), 50% hydrogen peroxide (0.33 mL, 11.529 mmol) was added at room temperature, and the reaction mixture was stirred for 24 h. The reaction was poured into water (20 mL) and extracted with hexane (5 × 30 mL). Then, the organic extract was washed with 10% $NaHCO_3$ (20 mL), dried with Na_2SO_4, concentrated under reduced pressure and purified by column chromatography using hexane as a mobile phase to obtain a colorless oil **3** (274 mg, 96%). TLC: hexane, R_f = 0.96. ^{13}C NMR (125 MHz, $CDCl_3$): δ = 113.94, 113.14, 31.10, 29.71, 28.72, 28.04, 27.76, 23.21, 14.10 ppm. IR: ν_{max} 2194, 2091, 1090, 711 cm^{-1}.

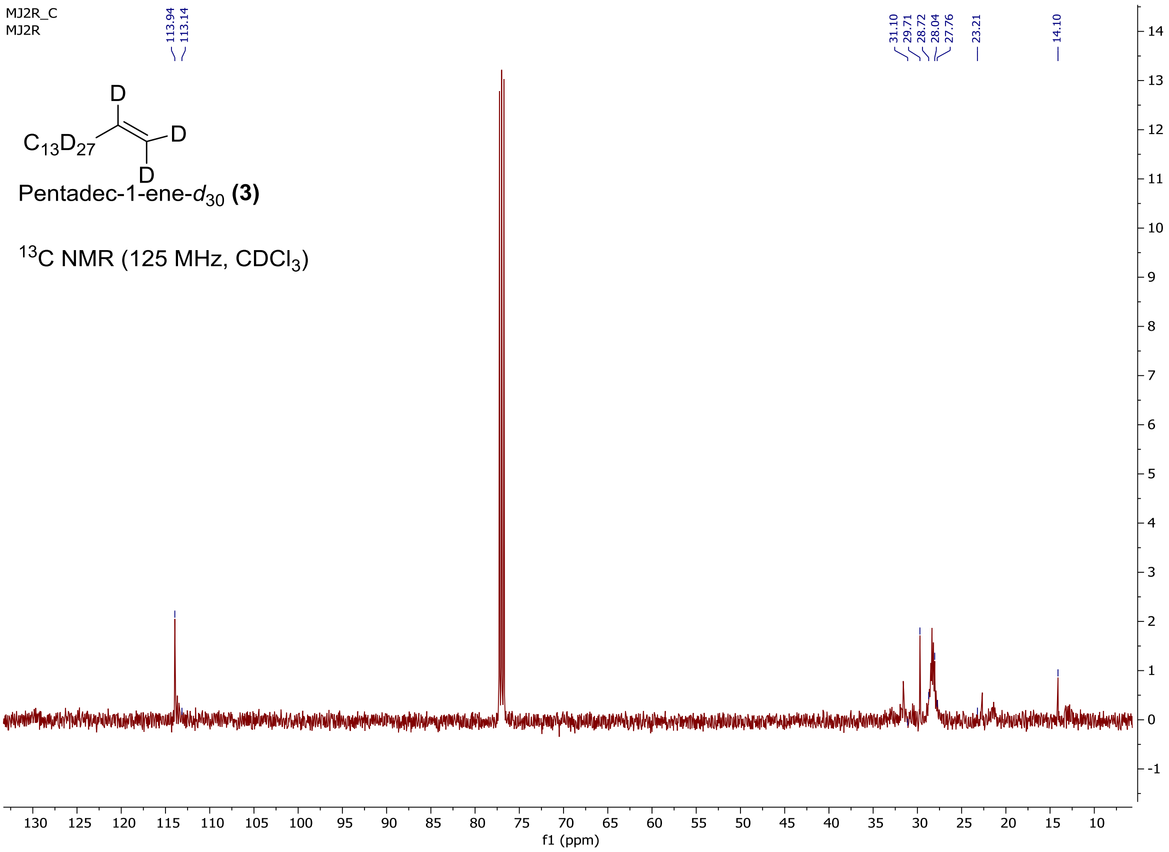

Tert-butyl (*S*)-4-((*R*)-1-hydroxyallyl)-2,2-dimetyloxazolidine-3-carboxylate (5)

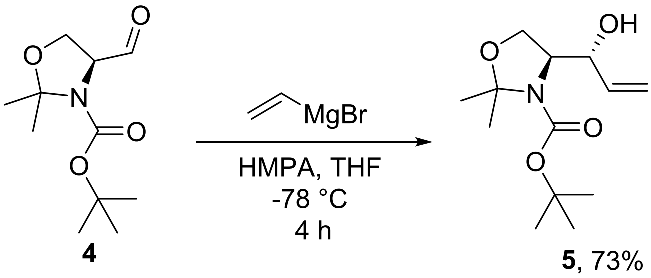

4 **5**, 73%

To a solution of (*S*)-Garner´s aldehyde **4** (444 mg, 1.937 mmol) in 4 mL of dry THF, a solution of vinylmagnesium bromide (1M in THF, 7.0 mL, 6.973 mmol) was dropwise added, followed by addition of hexamethylphosphoramide (HMPA, 0.46 mL, 2.617 mmol) under argon atmosphere at –78°C. After 2 h, the reaction mixture was slowly warmed to room temperature (approximately 2 h), then carefully quenched by saturated aqueous NH_4Cl (15 mL) and extracted with Et_2O (5 × 20 mL). The extract was dried with Na_2SO_4, concentrated under reduced pressure and purified by column chromatography using hexane/EtOAc (5:1) as a mobile phase to obtain a colorless oil **5** (328.3 mg, 73%). TLC: hexane/EtOAc (3:1), R_f = 0.34. ^{1}H NMR (500 MHz, $CDCl_3$): δ = 5.95 – 5.76 (m, 1H), 5.38 (dt, *J* = 17.1 Hz, 1.7 Hz, 1H), 5.22 (dt, *J* = 10.5 Hz, 1.6 Hz, 1H), 4.43 – 4.24 (m, 1H), 4.22 – 4.12 (m, 1H), 4.08 – 3.98 (m, 1H), 3.95 – 3.78 (m, 1H), 1.55 (s, 3H), 1.52 – 1.43 (m, 12H) ppm. ^{13}C NMR (125 MHz, $CDCl_3$): δ = 136.68, 115.47, 94.50, 81.29, 74.25, 64.73, 62.03, 28.33, 26.24, 24.46 ppm. IR: ν_{max} 2979, 1697, 1682, 1392, 1366, 1173, 1097, 848 cm^{-1}.

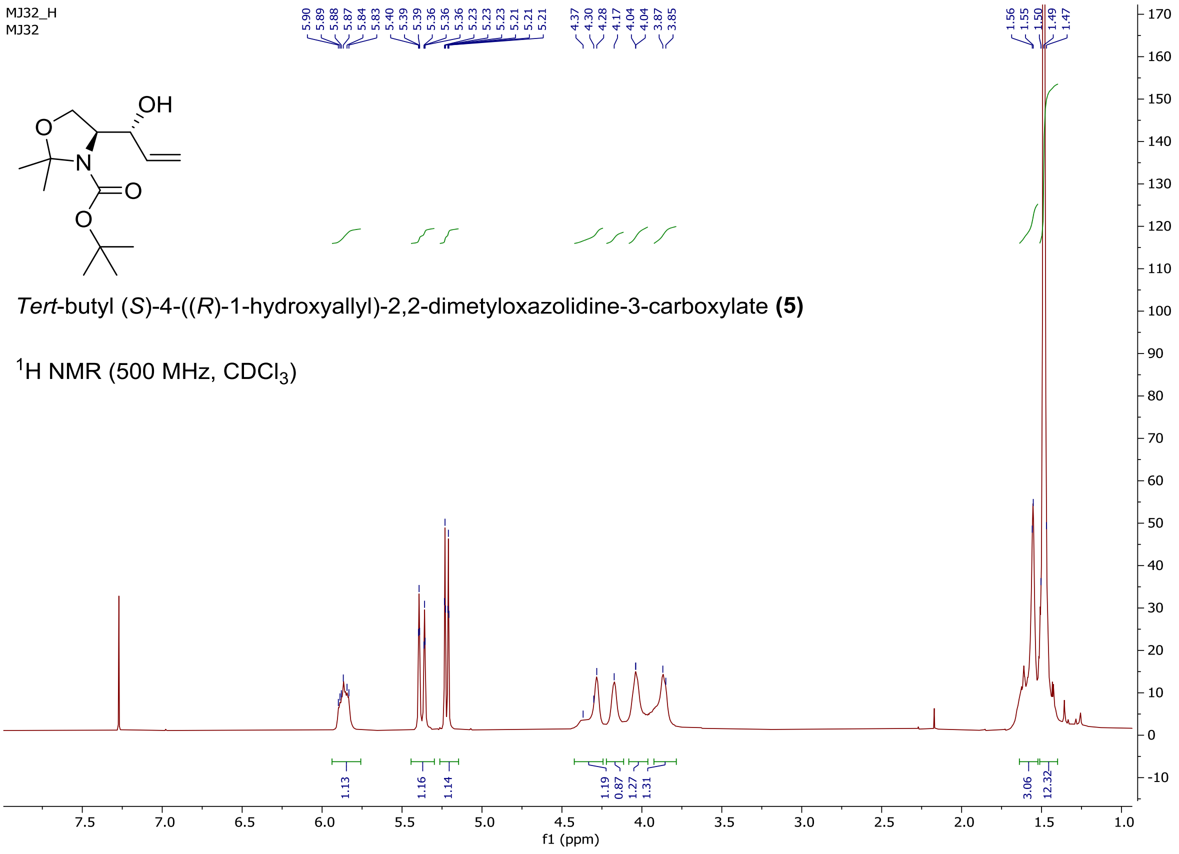

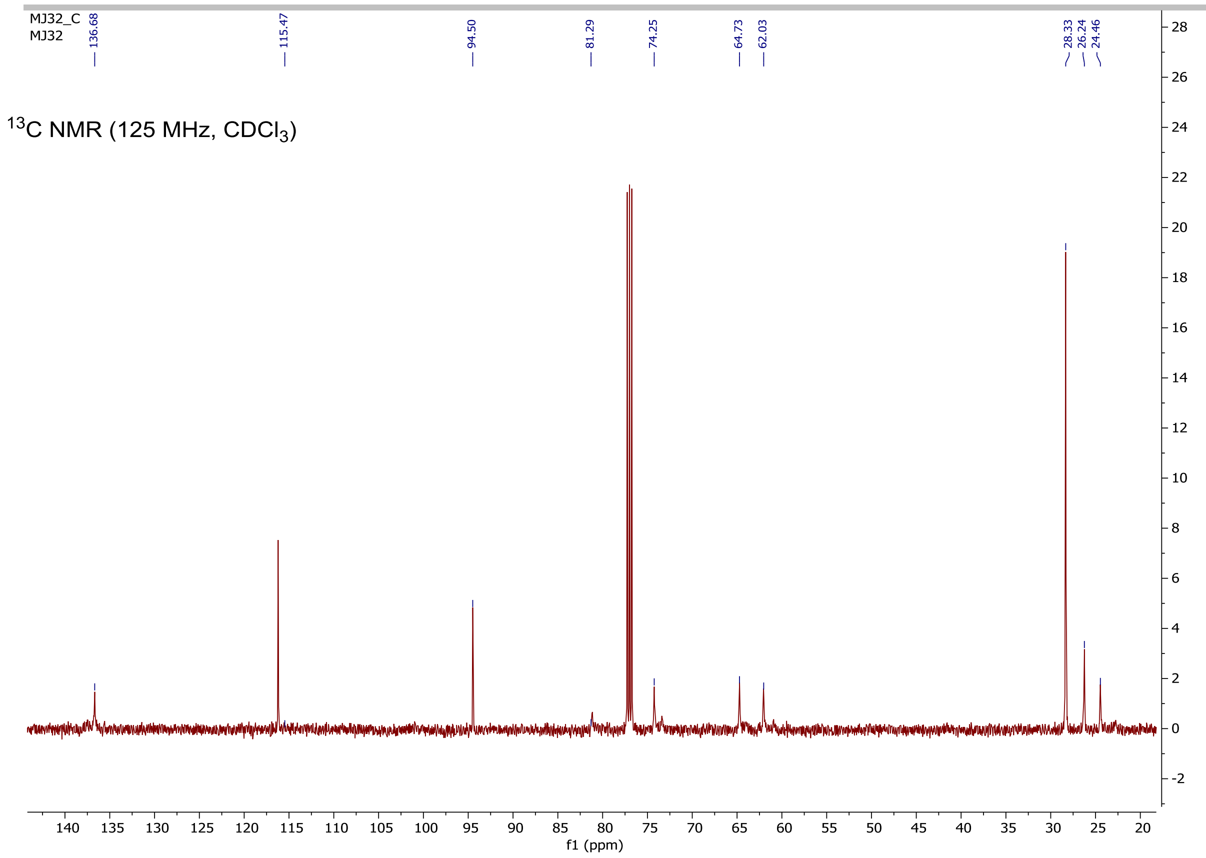

***Tert*-butyl (*S*)-4-((*R*,*E*)-1-hydroxyhexadec-2-ene-1-yl)-2,2-dimetyloxazolidine-3-carboxylate-*d*$_{28}$ (6)**

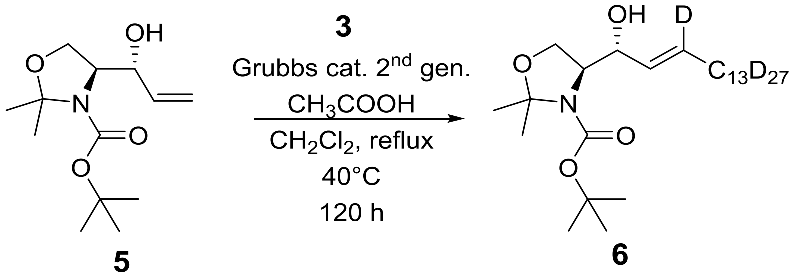

To a solution of intermediate **5** (35.4 mg, 0.138 mmol), alkene **3** (31.4 mg, 0.131 mmol), and Grubbs catalyst (3.5 mg, 0.013 mmol) in 0.5 mL of dry CH_2Cl_2, a dry CH_3COOH (2.25 µL, 0.039 mmol) was dropwise added. The reaction mixture was stirred at 40°C under argon atmosphere for 120 h. The reaction mixture was concentrated under reduced pressure and purified by column chromatography using hexane/EtOAc (9:1) as a mobile phase to obtain a colorless oil **6.** The reaction was repeated three times with 37±5% yields. TLC: hexane/EtOAc (3:1), R_f= 0.50. ^{1}H NMR (500 MHz, $CDCl_3$): δ = 5.44 (d, J = 6.2 Hz, 1H), 4.26 – 4.08 (m, 2H), 4.06 – 3.93 (m, 1H), 3.91 – 3.73 (m, 1H), 1.66 – 1.32 (m, 15H) ppm. ^{13}C NMR (125 MHz, $CDCl_3$): δ = 153.70, 128.02, 94.42, 81.00, 74.01, 64.90, 64.32, 62.30, 31.21, 29.73, 28.74, 28.36, 28.30, 28.07, 28.01, 26.24, 24.25, 23.70, 19.8 ppm. IR: ν_{max} 2197, 2095, 1698, 1391, 1089, 759 cm^{-1}.

MJ4R_H
MJ4R

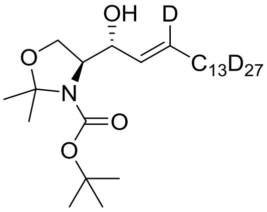

Tert-butyl (*S*)-4-((*R*,*E*)-1-hydroxyhexadec-2-ene-1-yl)-2,2-dimetyloxazolidine-3-carboxylate-d_{28} **(6)**

^{1}H NMR (500 MHz, $CDCl_3$)

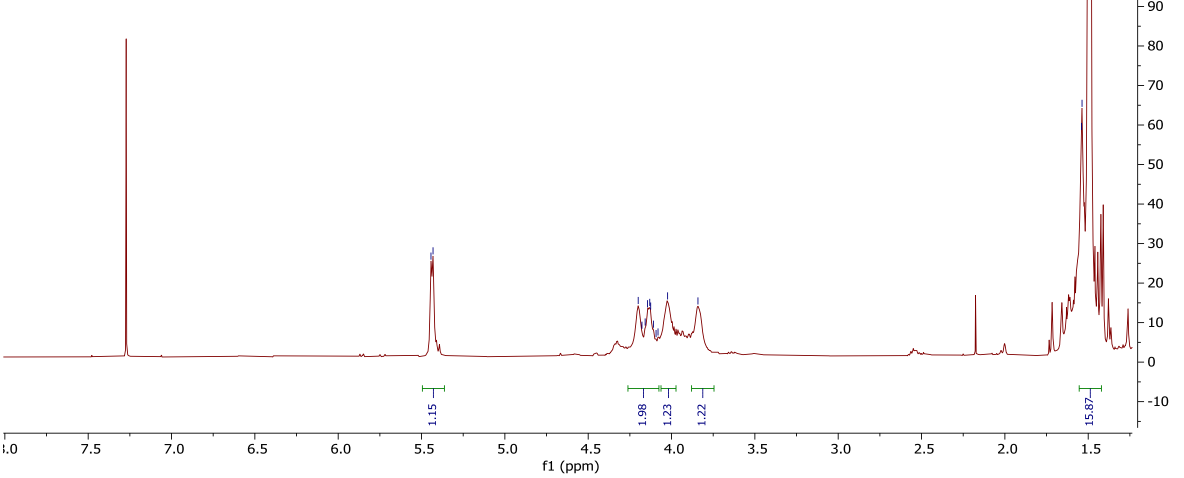

MJ4R_C
MJ4R

^{13}C NMR (125 MHz, $CDCl_3$)

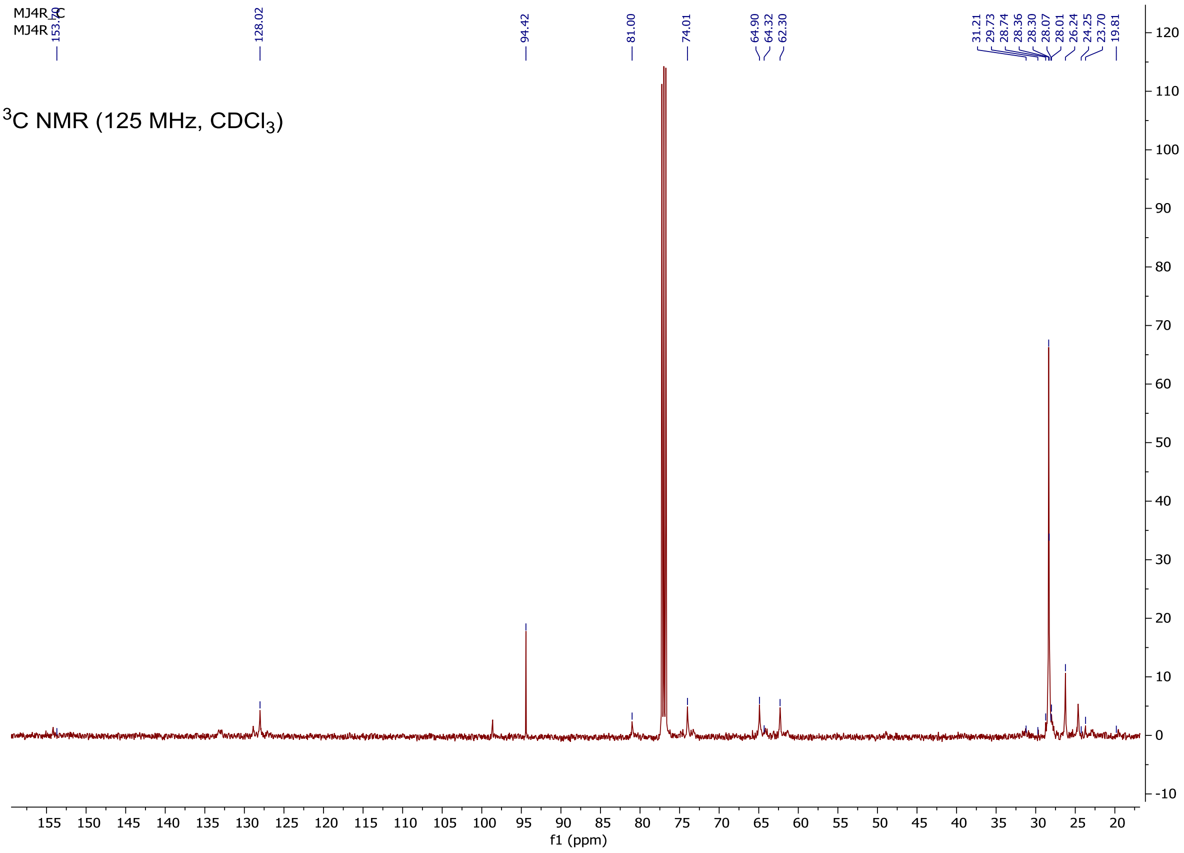

(2*S*,3*R*,4*E*)-*N*-Tetracosanoyl-2-aminooctadec-4-ene-1,3-diol-d_{28} (Cer[NS]-sph-d_{28})

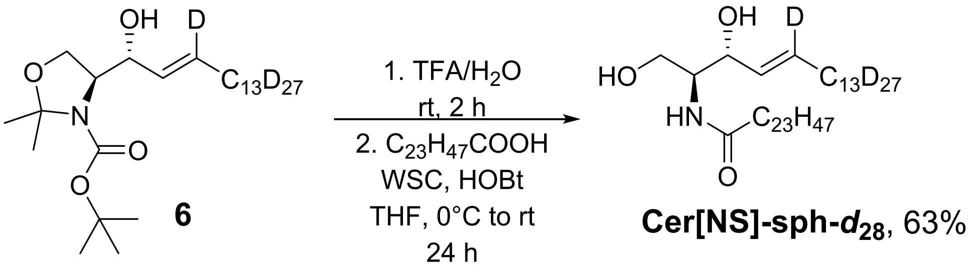

A solution of **6** (60 mg, 0.128 mmol) in a mixture of CF_3COOH/water (3:1, 2.75 mL) was stirred at room temperature for 2 h. The reaction mixture was neutralized with aqueous ammonia solution (pH ~ 8-9) and extracted with $CHCl_3$ (5 × 30 mL). The organic layer was dried with Na_2SO_4 and concentrated under reduced pressure to obtain 40.7 mg of sphingosine-d_{28}. TLC (crude product): BuOH/CH_3COOH/water (4:1:1), R_f = 0.52. LA (43.6 mg, 0.118 mmol) and 1-hydroxybenzotriazole (HOBt, 62.3 mg, 0.461 mmol) in dry THF (5 mL) were added to sphingosine-d_{28}, and 50 µL (0.249 mmol) WSC was added at 0°C (ice-cooled bath). The reaction mixture was stirred overnight at room temperature. The reaction was concentrated under reduced pressure and purified by column chromatography using $CHCl_3$/MeOH (50:1) as a mobile phase to obtain white crystals of **Cer[NS]-sph-d_{28}** (54.4 mg, 63%, two steps). TLC: $CHCl_3$/MeOH (10:1), R_f = 0.40. M. p.: 89–91°C. ^{1}H NMR (500 MHz, $CDCl_3$): 6.25 (d, *J* = 7.5 Hz, 1H), 5.53 (d, *J* = 6.5 Hz, 1H), 4.38 – 4.25 (m, 1H), 4.02 – 3.87 (m, 2H), 3.77 – 3.65 (m, 1H), 2.24 (t, *J* = 7.6 Hz, 2H), 1.72 – 1.54 (m, 2H), 1.46 – 1.09 (m, 40H), 0.89 (t, *J* = 6.9 Hz, 3H) ppm. ^{13}C NMR (125 MHz, $CDCl_3$) δ = 173.89, 134.21, 128.71, 74.67, 62.52, 54.48, 36.84, 31.91, 29.69, 29.28, 25.75, 23.15, 14.10 ppm. MS (APCI$^+$): *m/z* 679.0 (M+H$^+$). Elemental analysis: $C_{42}H_{55}D_{28}NO_3$ (678.30) Calcd.: C, 74.37; H, 16.49; O, 7.08; N, 2.07; found: C, 73.99; H, 16.83; O, 7.09; N, 2.09. IR: ν_{max} 2918, 2850, 2194, 2089, 1640, 1555, 1467, 802, 729 cm^{-1}.

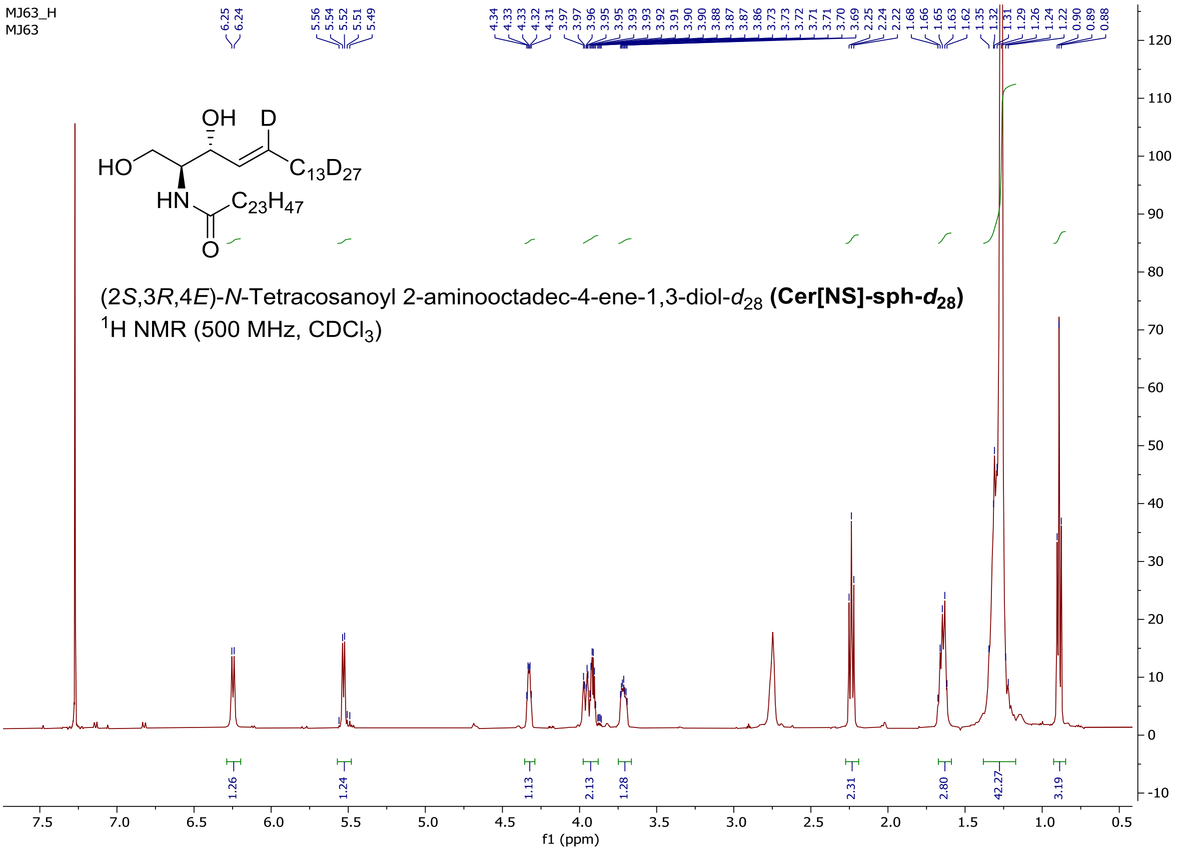

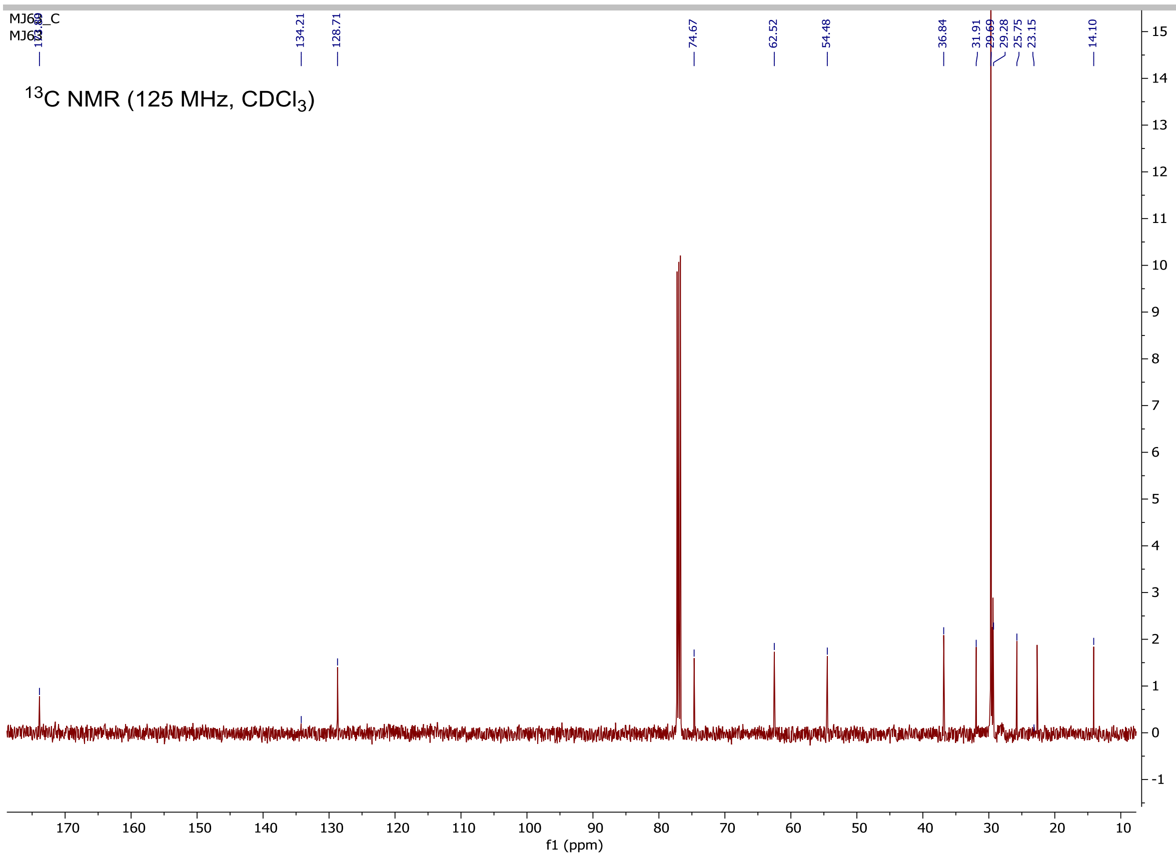

(2-Nitrophenyl)(pentadecyl)selane (2a)

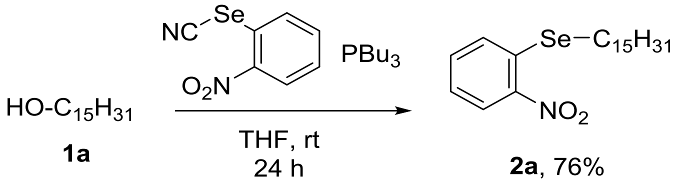

To a solution of pentadecanol **1a** (678 mg, 2.97 mmol) and 2-nitrophenyl selenocyanate (800 mg, 3.52 mmol) in dry THF (25 mL), tributylphosphine (0.87 mL, 3.49 mmol) was added and the reaction mixture was stirred under argon atmosphere at room temperature overnight. The reaction mixture was then concentrated under reduced pressure and purified by column chromatography using hexane/Et_2O (10:1) as a mobile phase to obtain yellow crystals **2a** (934 mg, 76%). TLC: hexane/Et_2O (3:1), R_f = 0.71. M. p. 51–55°C. ^{1}H NMR (500 MHz, $CDCl_3$): δ = 8.32 – 8.27 (m, 1H), 7.56 – 7.49 (m, 2H), 7.33 – 7.28 (m, 1H), 2.96 – 2.89 (m, 2H), 1.84 – 1.74 (m, 2H), 1.53 – 1.45 (m, 2H), 1.40 – 1.20 (m, 22H), 0.89 (t, J = 6.9 Hz, 3H) ppm. ^{13}C NMR (125 MHz, $CDCl_3$): δ = 146.89, 133.96, 133.41, 129.02, 126.42, 125.16, 31.90, 30.15, 29.67, 29.66, 29.64, 29.61, 29.56, 29.45, 29.34, 29.13, 28.28, 26.29, 26.02, 22.67, 14.09 ppm. IR: ν_{max} 2915, 2850, 2360, 2342, 1509, 1471, 1332, 1307, 1098, 1039, 720, 669 cm^{-1}.

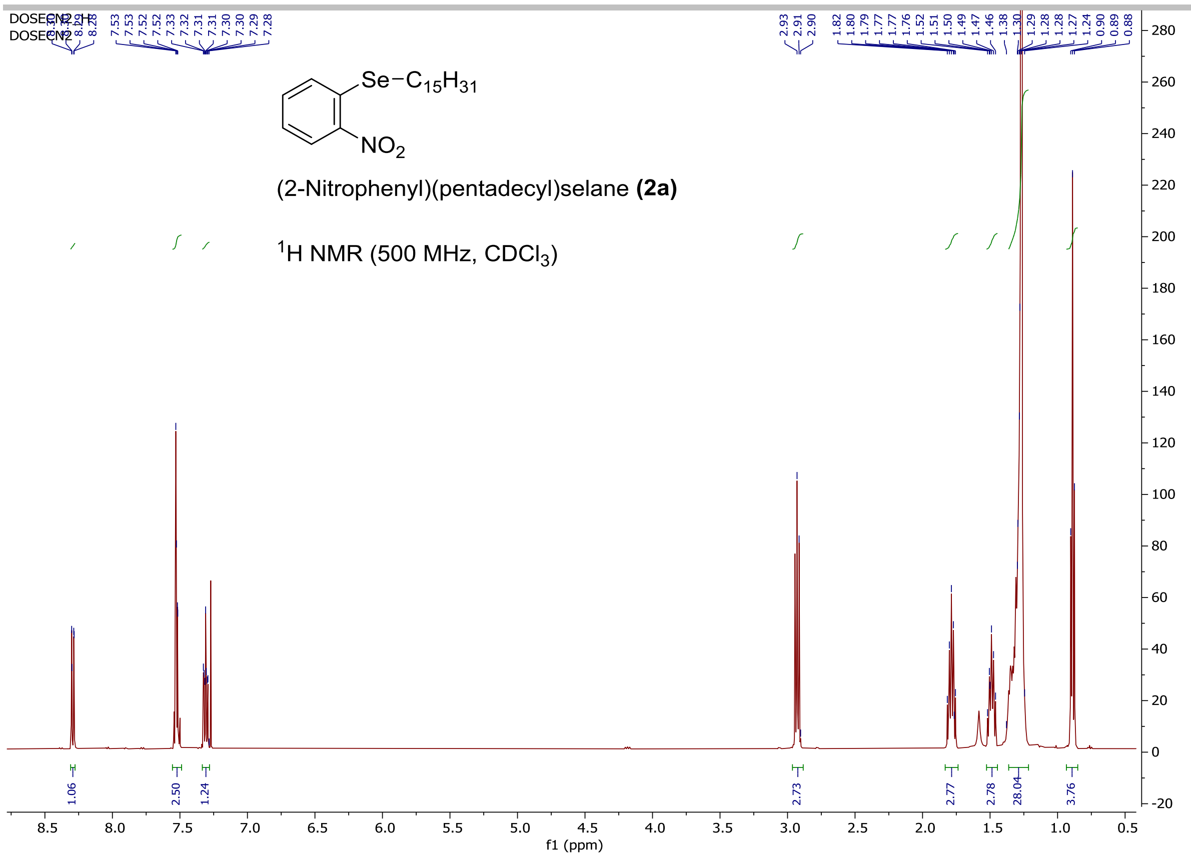
Se−C15H31
NO2
(2-Nitrophenyl)(pentadecyl)selane **(2a)**
1H NMR (500 MHz, CDCl3)
f1 (ppm)

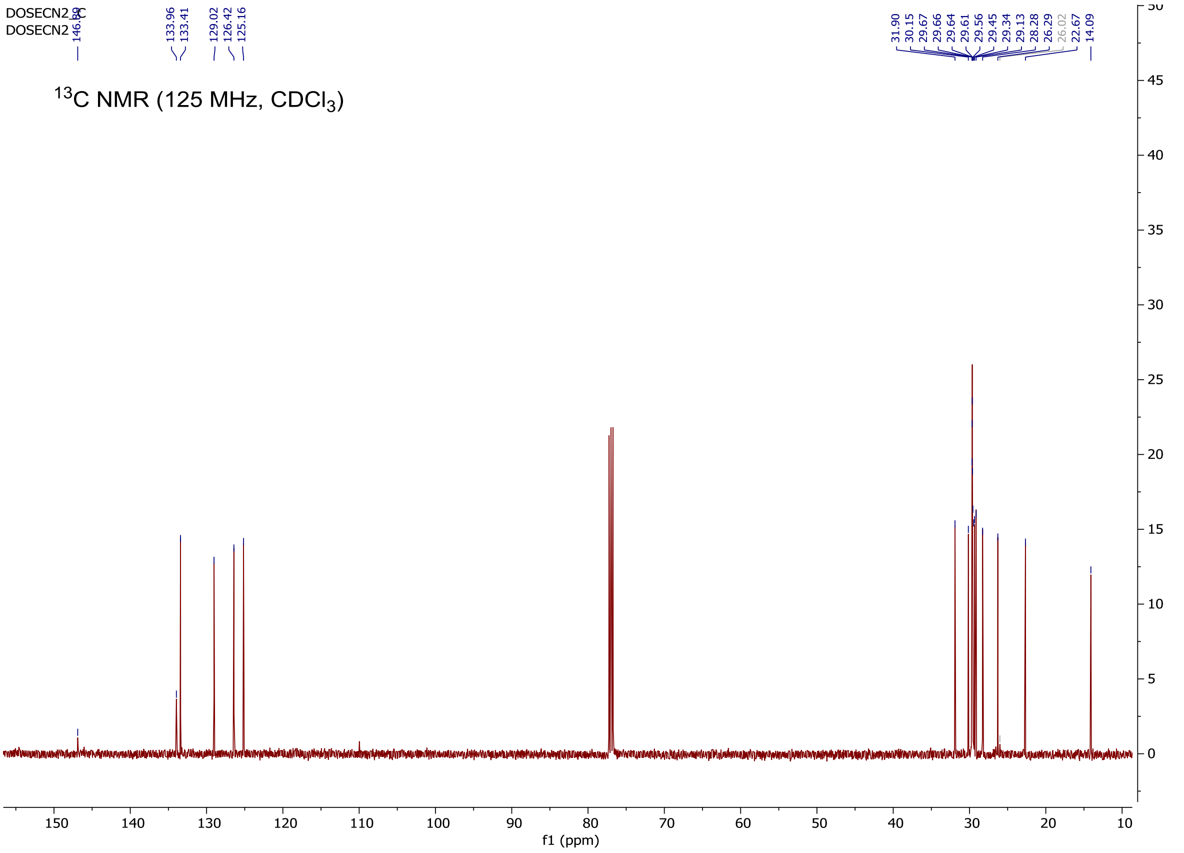
13C NMR (125 MHz, CDCl3)
f1 (ppm)

Pentadec-1-ene (3a)

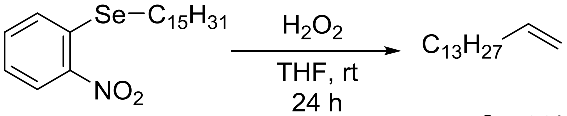

2a **3a**, 96%

To a solution of **2a** (934 mg, 2.264 mmol) in dry THF (30 mL), 50% hydrogen peroxide (0.7 mL, 22.643 mmol) was added at room temperature, and the reaction mixture was stirred for 24 h. The reaction was poured into water (20 mL) and extracted with hexane (5 × 30 mL). Then, the organic extract was washed with 10% $NaHCO_3$ (20 mL), dried with Na_2SO_4, concentrated under reduced pressure and purified by column chromatography using hexane as a mobile phase to obtain a colorless oil **3a** (450 mg, 96%). TLC: hexane, R_f= 0.96. ^{1}H NMR (500 MHz, $CDCl_3$): δ = 5.88 – 5.78 (m, 1H), 5.04 – 4.97 (m, 1H), 4.96 – 4.90 (m, 1H), 2.09 – 2.01 (m, 2H), 1.43 – 1.34 (m, 2H), 1.34 – 1.22 (m, 20H), 0.89 (t, *J* = 7.0 Hz, 3H) ppm. ^{13}C NMR (125 MHz, $CDCl_3$): δ = 139.26, 114.04, 33.82, 31.92, 29.69, 29.67, 29.65, 29.61, 29.50, 29.35, 29.15, 28.95, 22.68, 14.11 ppm. IR: ν_{max} 2923, 2853, 2360, 2341, 1489, 991, 909 cm^{-1}.

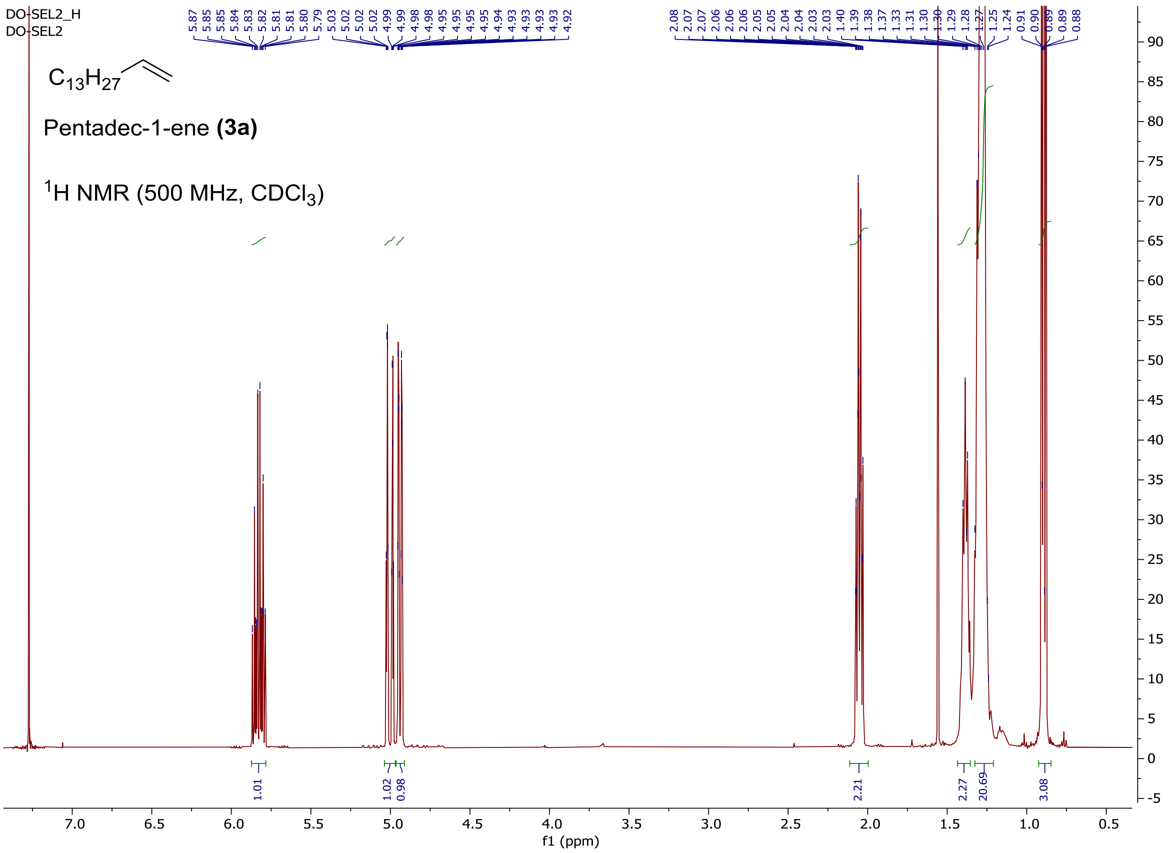

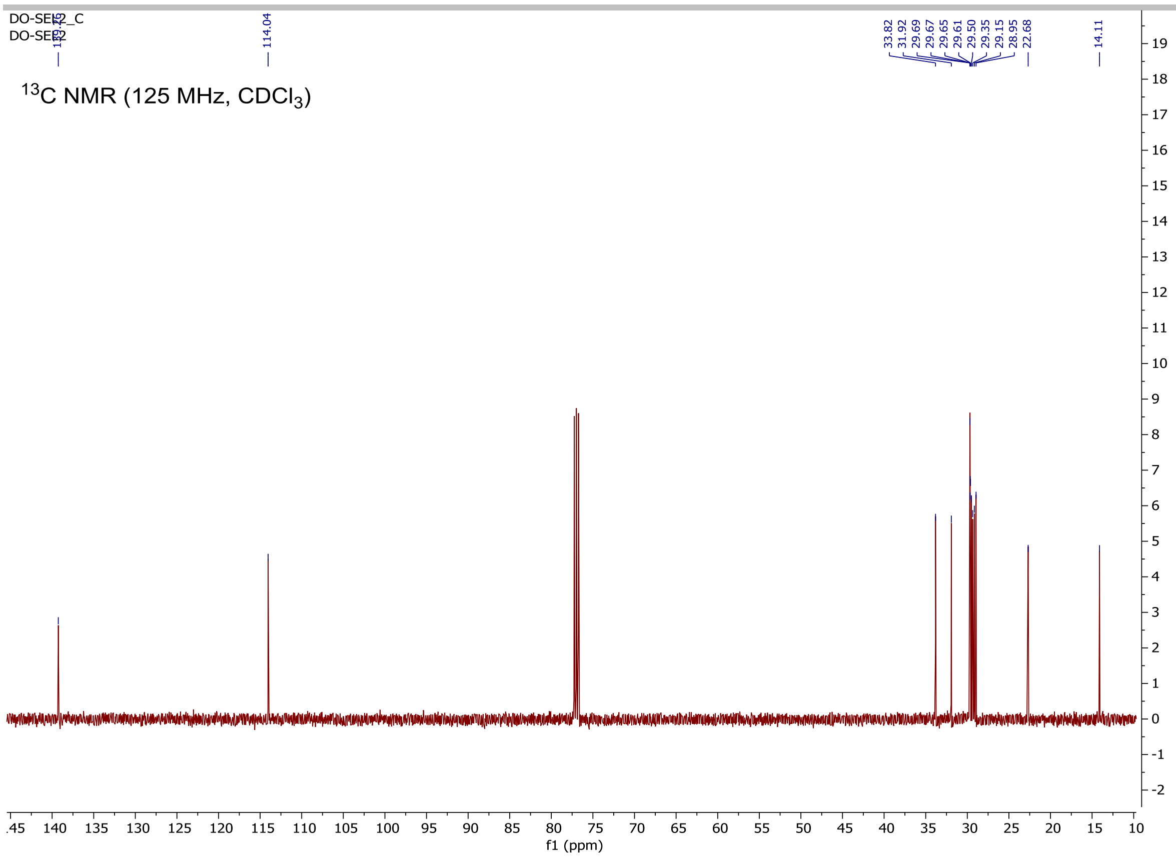

Tert-butyl (*S*)-4-((*R*,*E*)-1-hydroxyhexadec-2-ene-1-yl)-2,2-dimetyloxazolidine-3-carboxylate (6a)

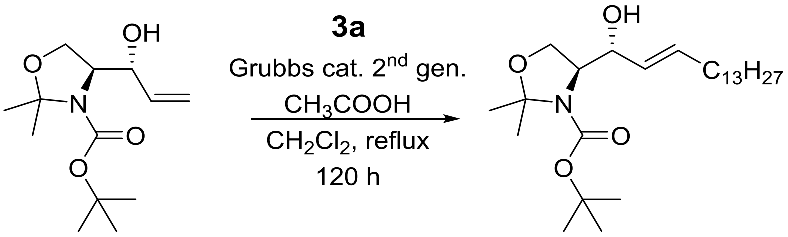

5 **6a**, 59%

To a solution of intermediate **5a** (50 mg, 0.194 mmol), alkene **3a** (41 mg, 0.194 mmol), and Grubbs catalyst (3.5 mg, 0.004 mmol) in 0.25 mL of dry CH_2Cl_2, a dry CH_3COOH (2.25 µL, 0.039 mmol) was dropwise added. The reaction mixture was stirred at 40°C under argon atmosphere for 120 h. The reaction mixture was concentrated under reduced pressure and purified by column chromatography using hexane/EtOAc (9:1) as a mobile phase to obtain a colorless oil **6a**. The reaction was repeated three times with 57±1% yields. TLC: hexane/EtOAc (3:1), R_f = 0.50. ^{1}H NMR (500 MHz, $CDCl_3$): δ = 5.78 – 5.68 (m, 1H), 5.44 (dd, J = 15.5, 6.2 Hz, 1H), 4.25 – 4.17 (m, 1H), 4.15 – 4.08 (m, 1H), 4.06 – 3.96 (m, 1H), 3.87 – 3.77 (m, 1H), 2.09 – 2.00 (m, 2H), 1.56 – 1.51 (m, 2H), 1.51 – 1.45 (m, 12H), 1.42 – 1.32 (m, 2H), 1.32 – 1.20 (m, 21H), 0.92 – 0.84 (m, 3H) ppm. ^{13}C NMR (125 MHz, $CDCl_3$): δ = 154.14, 133.37, 128.09, 94.39, 80.98, 74.00, 64.89, 64.16, 62.28, 32.37, 31.89, 29.65, 29.64, 29.62, 29.57, 29.47, 29.32, 29.28, 29.21, 29.11, 28.34, 26.23, 24.61, 22.62, 14.08 ppm. IR: ν_{max} 2923, 2853, 1702, 1693, 1384, 1366, 1172, 1096 cm^{-1}.

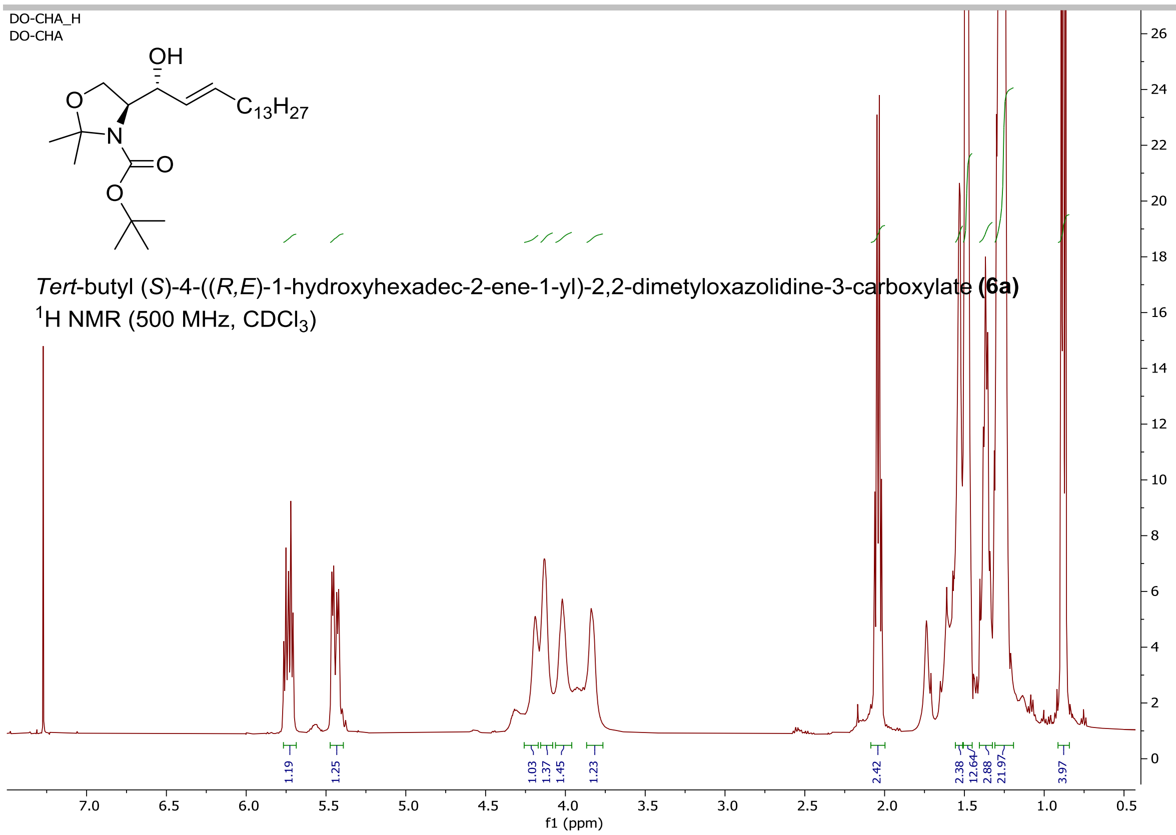
DO-CHA_H
DO-CHA
OH
O
N
O
O
$C_{13}H_{27}$
Tert-butyl (*S*)-4-((*R,E*)-1-hydroxyhexadec-2-ene-1-yl)-2,2-dimetyloxazolidine-3-carboxylate **(6a)**
^{1}H NMR (500 MHz, $CDCl_3$)
1.19
1.25
1.03
1.37
1.45
1.23
2.42
2.38
12.64
2.88
21.97
3.97
f1 (ppm)

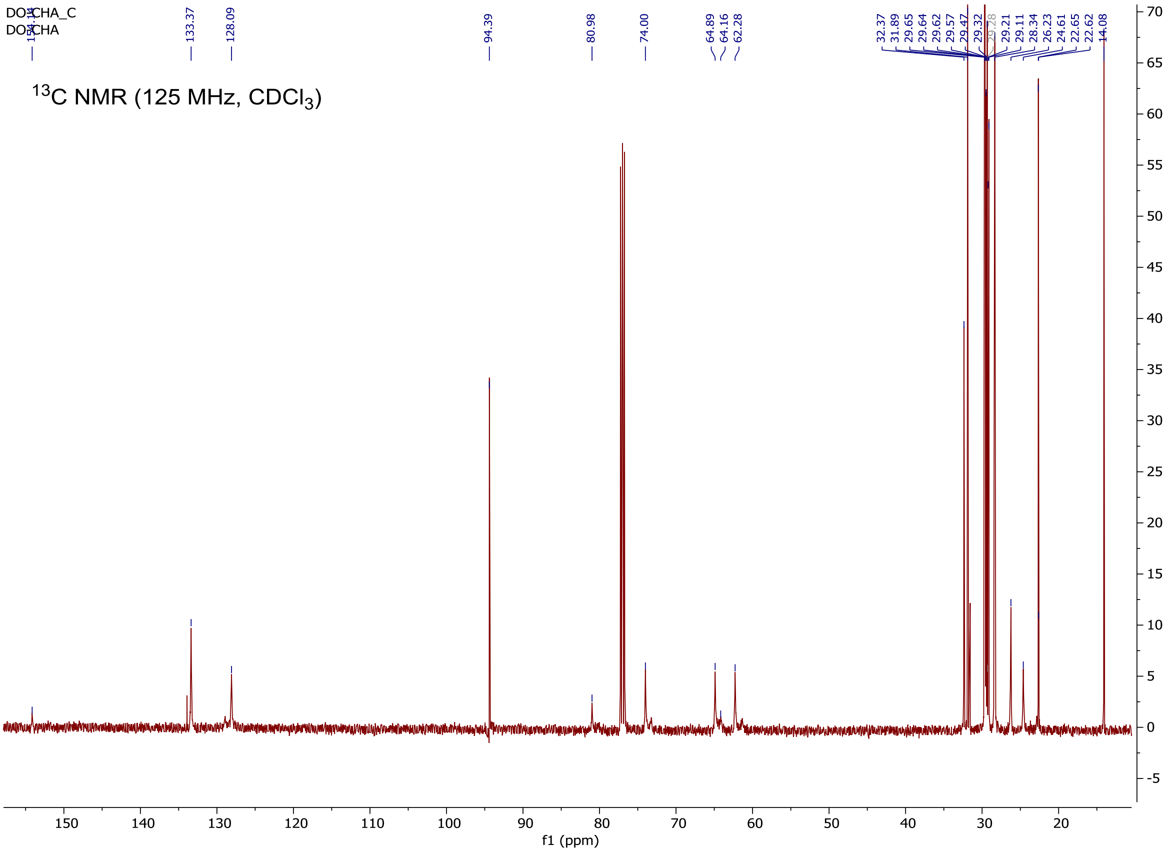
DO-CHA_C
DO-CHA
133.37
128.09
94.39
80.98
74.00
64.89
64.16
62.28
32.37
31.89
29.65
29.64
29.62
29.57
29.47
29.32
29.21
29.11
28.34
26.23
24.61
22.65
22.62
14.08
^{13}C NMR (125 MHz, $CDCl_3$)
f1 (ppm)

1.4 Sample preparation

Aliquots of Cer[NS], LA, and cholesterol were co-dissolved in $CHCl_3$/MeOH (2:1) at molar ratios of 1:1:1 and 1:1:0.45. The organic solvent was evaporated at 40°C under vacuum in a rotary evaporator followed by redissolving the lipid film in cyclohexane and freezing in liquid nitrogen. Overnight lyophilization resulted in a fluffy powder, that was hydrated to 50 wt% in aqueous buffer (100 mM 2-(*N*-morpholino)ethanesulfonic acid (MES), 100 mM NaCl, 5 mM EDTA, pH 5.4, prepared in deuterium-depleted water). The sample was equilibrated by repeatedly heating to 80°C and freezing in liquid nitrogen for 10 times. To provide an inert environment and prevent sample dehydration, the samples were filled into 4 mm MAS rotors and slowly cooled to room temperature. To prevent kinetic effects on phase formations,[2] the samples were incubated at room temperature for 24 hours before the NMR measurements.

1.5 Solid-state ^{2}H NMR spectroscopy

A wide bore Bruker Avance 750 MHz NMR spectrometer operating at a resonance frequency of 115.1 MHz was used to acquire the ^{2}H NMR spectra of the SC samples. A single channel solids probe with a 5 mm solenoid coil was used. Spectrum acquisition was performed using a phase-cycled quadrupolar echo sequence[3] with two π/2 pulses of approximately 2.4 µs length separated by a 30 µs delay. The spectral width was ±250 kHz. The relaxation delay was set to 50 s, because the relaxation of ^{2}H in a rigid phase can be up to 10 s.[4] The ^{2}H NMR spectra of all samples were acquired at temperatures of 25, 32, 50, 65, and 80°C. To quantify the individual phases observed in the NMR spectra, numerical simulation were performed using a program written in Mathcad (MathSoft, Cambridge, MA) as previously described.[5] For the rigid crystalline phase, a maximum quadrupolar splitting of 125.25 kHz was used for n C-D bonds corresponding to a quadrupolar coupling constant of 167 kHz. Under such conditions, the methyl groups would be free to rotate resulting in a scaling of the quadrupolar splitting by a factor of 1/3, corresponding to a Pake doublet of 41.75 kHz width for the terminal methyl groups. The NMR spectra of the ^{2}H labeled sphingosine moiety at temperatures of 25°C and 32°C showed a sizable fraction of a lineshape that would suggest two-site-exchange motions.[6] Accordingly, the ^{2}H NMR spectrum was simulated using a hop angle of 120° using NMR-WEBLAB.[7] At higher temperatures, the ^{2}H NMR spectra were simulated assuming a superposition of ^{2}H NMR Pake doublets, scaled by the appropriate order parameter (S_{CD}). An order parameter gradient was assumed with highest molecular order at the beginning of the chain and decreasing order towards the end of the chain as known from the ^{2}H NMR line shapes of phospholipids in the liquid ordered phase state. [8-10]A single Lorentzian line was used to simulate the isotropic phase.

1.6 X-Ray diffraction

After ^{2}H NMR spectroscopy measurements, the same samples were transferred from the MAS rotors onto a cover glass 22 × 22 mm^2 mounted in the modified XRD sample holder for samples on substrates with the inner diameter of 32 mm. The XRD data were collected at ambient room temperature and humidity with an X'Pert PRO θ-θ diffractometer (PANalytical B.V., Almelo, The Netherlands) with parafocusing Bragg-Brentano geometry using CoKα radiation (λ = 1.7903 Å, U = 35 kV, I = 40 mA) over the angular range of 0.6–30° (2θ). Data were scanned with an ultrafast position-sensitive linear (1D) X'Celerator detector with a step size of 0.0167° (2θ) and a counting time of 20.32 s.step^{-1}. The data were evaluated using X'Pert Data Viewer software (PANalytical B.V., Almelo, The Netherlands). The XRD patterns show the raw scattered intensities as a function of the scattering vector *q* (Å^{-1}), which is proportional to the scattering angle 2θ according to the equation: $q = 4\pi \sin\theta/\lambda$ (λ is a wavelength of the X-rays). The repeat distance *d* (Å) characterizes the spacing between adjacent scattering planes for

parallel lipid layers arranged in a 1D lattice. The XRD patterns of the lamellar phases exhibit a set of Bragg reflections with reciprocal spacings in the characteristic ratios of $q_n = 2\pi n/d$ (Miller index n = 1, 2, 3…). The d was obtained from the slope a of a linear regression of the dependence $q_n = a.n + q_0$, according to the equation $d = 2\pi/a$, where q_0 is a constant corresponding to a shift of the origin.

1.7 Infrared spectroscopy

Cer[NS]-sph-d_{28} or Cer[NS]-acyl-d_{47}, LA, Chol, and cholesteryl sulfate were dissolved in 2:1 hexane/96% EtOH (v/v) (cholesteryl sulfate was dissolved in 96% aq. EtOH) and mixed at 1:1:1:0.13 molar ratios. These lipid solutions were evaporated under stream of nitrogen and then heated to 90°C, a temperature that is above the main phase transitions of all the studied samples, equilibrated for 10 min, and then slowly (~3 h) cooled to room temperature. Then, the samples were equilibrated at 32°C and 30% air humidity for 24 h. Infrared spectra of the samples were collected on a Nicolet 6700 spectrometer (Thermo Scientific, USA) equipped with a single-reflection MIRacle ATR ZnSe crystal (PIKE technologies, Madison, USA). A clamping mechanism with constant pressure was used. The spectra were generated by the co-addition of 256 scans collected at a resolution of 2 cm^{-1}. The temperature dependence of the FTIR spectra was studied over the range of 28–100°C with 2°C steps using a temperature control module (PIKE technologies, Madison, USA).

2 Results

2.1 Supplementary Figure S1

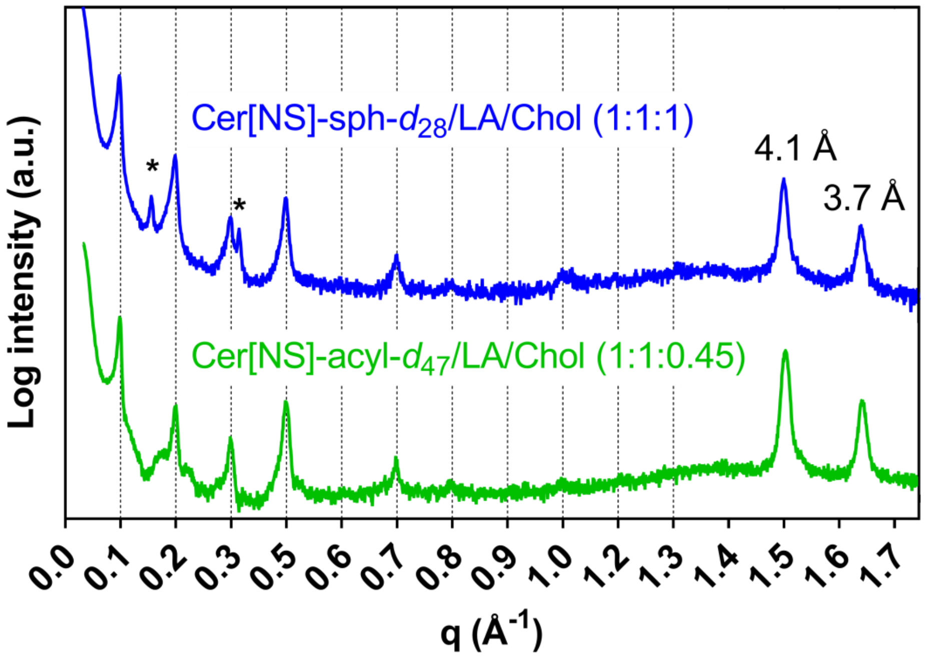

Supplementary Figure S1. X-ray diffraction pattern of SC lipid model mixture composed of Cer[NS], LA, and Chol at molar ratios of 1:1:0.45 (green) and 1:1:1 (blue) at room temperature.

2.2 Supplementary Figure S2

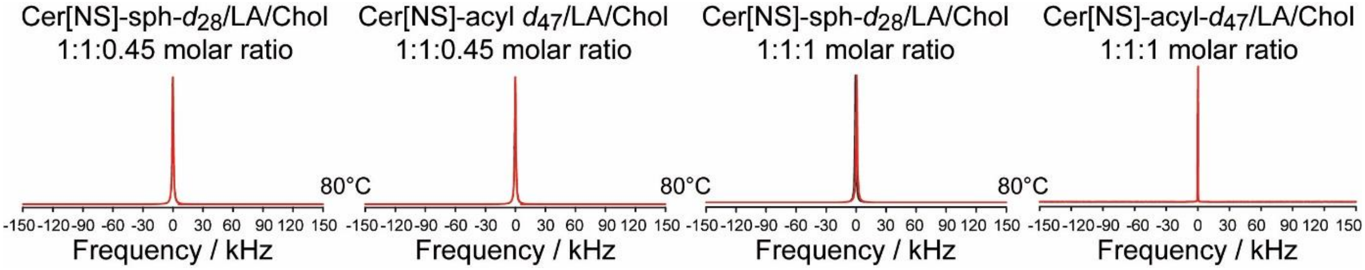

Supplementary Figure S2. Static ^2H NMR spectra (black) of the SC lipid model mixture composed of Cer[NS], LA, and Chol at molar ratios of 1:1:0.45 (left two columns) and 1:1:1 (right two columns) at 80°C. The first and the third column display the ^2H NMR spectra of the Cer[NS]-sph-d_{28} (perdeuterated sphingosine-d_{28}). Columns 2 and 4 show the ^2H NMR spectra of the Cer[NS]-acyl-d_{47} (perdeuterated lignoceroyl-d_{47}). Simulated spectra are shown in red. The multilamellar lipid preparations contained 50 wt% aqueous buffer (100 mM MES, 100 mM NaCl, 5 mM EDTA, pH 5.4). In this isotropic phase, the multilayer structure of the SC model found at lower temperatures is completely abolished. However, this phase restructuring is completely reversible and lamellar structures are formed again when the temperature is decreases.

2.3 Supporting Table S1

Supporting Table S1. Phase composition in model membranes composed of Cer[NS]/LA/Chol (1:1:0.45 and 1:1:1 molar ratio) as determined from numerical simulation of the ^{2}H NMR spectra shown in Figures 2 and S2.

	**Cer[NS]-sph-d_{28}/LA/Chol 1:1:0.45 (mol/mol/mol)**				**Cer[NS]-acyl-d_{47}/LA/Chol 1:1:0.45 (mol/mol/mol)**		
Temperature (°C)	cryst. (%)	2-site ex (%)	fluid (%)	isotropic (%)	cryst.(%)	fluid (%)	isotropic (%)
25	24	45	31	0	98	0	2
32	0	74	26	0	98	0	2
50	0	0	100	0	53	41	6
65	0	0	98	2	25	31	44
80	0	0	0	100	0	0	100
	**Cer[NS]-sph-d_{28}/LA/Chol 1:1:1 (mol/mol/mol)**				**Cer[NS]-acyl-d_{47}/LA/Chol 1:1:1 (mol/mol/mol)**		
Temperature (°C)	cryst. (%)	2-site ex (%)	fluid (%)	isotropic (%)	cryst. (%)	fluid (%)	isotropic (%)
25	0	73	26	1	86	13	1
32	0	68	32	0	84	15	1
50	0	0	99	1	6	93	1
65	0	0	49	51	4	65	31
80	0	0	0	100	0	2	98

3 References

[1] A. Kovacik, A. Vogel, J. Adler, P. Pullmannova, K. Vavrova, D. Huster, *Biochim. Biophys. Acta Biomembr.* **2018,** *1860*, 1162-1170.

[2] R. Mendelsohn, I. Selevany, D. J. Moore, M. C. Mack Correa, G. Mao, R. M. Walters, C. R. Flach, *J. Phys. Chem. B* **2014,** *118*, 4378-4387.

[3] J. H. Davis, K. R. Jeffrey, M. Bloom, M. I. Valic, T. P. Higgs, *Chem. Phys. Lett.* **1976,** *42*, 390-394.

[4] N. Kitson, J. Thewalt, M. Lafleur, M. Bloom, *Biochemistry* **1994,** *33*, 6707-6715.

[5] S. Stahlberg, B. Skolova, P. K. Madhu, A. Vogel, K. Vavrova, D. Huster, *Langmuir.* **2015,** *31*, 4906-4915.

[6] A. G. Palmer III, J. Williams, A. McDermott, *J. Phys. Chem.* **1996,** *100*, 13293-13310.

[7] V. Macho, L. Brombacher, H. W. Spiess, *Appl. Magn. Reson.* **2001,** *20*, 405-432.

[8] A. Bunge, P. Müller, M. Stöckl, A. Herrmann, D. Huster, *Biophys. J.* **2008,** *94*, 2680-2690.

[9] S. L. Veatch, O. Soubias, S. L. Keller, K. Gawrisch, *Proc. Natl. Acad. Sci. U. S. A.* **2007,** *104*, 17650-17655.

[10] T. Bartels, R. S. Lankalapalli, R. Bittman, K. Beyer, M. F. Brown, *J. Am. Chem. Soc.* **2008,** *130*, 14521-14532.

Author Contributions

D.H. and K.V. designed the research, M.J., A.K and L.O. synthesized the Cer[NS] lipids, A.K. carried out and analyzed the FTIR measurements, O.E. carried out the ^{2}H NMR experiments, D.H. and O.E. analyzed the NMR data and did the lineshape simulations, P.P. analyzed the X-ray diffraction experiments, D.H. wrote the paper with the contributions from all coauthors.
